# Adaptation of a Bioinformatics Microarray Analysis Workflow for a Toxicogenomic Study in Rainbow Trout

**DOI:** 10.1371/journal.pone.0128598

**Published:** 2015-07-17

**Authors:** Sophie Depiereux, Bertrand De Meulder, Eric Bareke, Fabrice Berger, Florence Le Gac, Eric Depiereux, Patrick Kestemont

**Affiliations:** 1 Unit of research in Environmental and Evolutionary Biology (URBE-NARILIS), Laboratory of Ecophysiology and Ecotoxicology, University of Namur, Namur, Belgium; 2 Unit of Research in Molecular Biology (URBM-NARILIS), University of Namur, Namur, Belgium; 3 Institut National de la Recherche Agronomique, INRA-LPGP, UPR1037, Campus de Beaulieu, 35042, Rennes, France; 4 Sainte-Justine UHC Research Centre, University of Montreal, Montréal (Québec), H3T 1C5, Canada; Temasek Life Sciences Laboratory, SINGAPORE

## Abstract

Sex steroids play a key role in triggering sex differentiation in fish, the use of exogenous hormone treatment leading to partial or complete sex reversal. This phenomenon has attracted attention since the discovery that even low environmental doses of exogenous steroids can adversely affect gonad morphology (ovotestis development) and induce reproductive failure. Modern genomic-based technologies have enhanced opportunities to find out mechanisms of actions (MOA) and identify biomarkers related to the toxic action of a compound. However, high throughput data interpretation relies on statistical analysis, species genomic resources, and bioinformatics tools. The goals of this study are to improve the knowledge of feminisation in fish, by the analysis of molecular responses in the gonads of rainbow trout fry after chronic exposure to several doses (0.01, 0.1, 1 and 10 μg/L) of ethynylestradiol (EE2) and to offer target genes as potential biomarkers of ovotestis development. We successfully adapted a bioinformatics microarray analysis workflow elaborated on human data to a toxicogenomic study using rainbow trout, a fish species lacking accurate functional annotation and genomic resources. The workflow allowed to obtain lists of genes supposed to be enriched in true positive differentially expressed genes (DEGs), which were subjected to over-representation analysis methods (ORA). Several pathways and ontologies, mostly related to cell division and metabolism, sexual reproduction and steroid production, were found significantly enriched in our analyses. Moreover, two sets of potential ovotestis biomarkers were selected using several criteria. The first group displayed specific potential biomarkers belonging to pathways/ontologies highlighted in the experiment. Among them, the early ovarian differentiation gene *foxl2a* was overexpressed. The second group, which was highly sensitive but not specific, included the DEGs presenting the highest fold change and lowest p-value of the statistical workflow output. The methodology can be generalized to other (non-model) species and various types of microarray platforms.

## Introduction

### 2.1 Intersex in wild fish exposed to ethynylestradiol

The issue of increased reproductive failure and intersexuality in wild fish due to the release of endocrine disrupting compounds (EDCs) in surface waters by sewage has attracted much attention in the scientific community over the last 20 years. Of great concern is the ability of very low doses (in the range of ng to μg/L) of xenoestrogenic substances (molecules that mimic the natural estradiol) to alter gonad morphology, typically manifesting as the development of oocytes within a functional testis (ovotestis) [1–3]. This feature has been correlated with reproductive dysfunctionalities and decreased population fitness in several fish species [4,5]. The impact of these compounds varies depending on the potency of the molecule, the synthetic estrogen ethynylestradiol (EE2) being in vivo the most potent [6] among the different EDCs, the duration of exposure and the species sensitivity. Many studies have reported testis-ova development and the phenomenon has been observed in all industrialized countries [7,8], but few studies have tackled the mechanisms underlying this phenomenon. Testis-ova and morphological disruptions are effective criteria that can be used to identify exogenous estrogen exposure [9]. However, their detection requires laborious histological analysis, with a limited area for investigation in the gonads, which thus renders the analysis unsuitable for quantitative evaluations. In this context, the use of marker genes expressed in correlation with testis-ova development may provide early signature of gonad morphological disruptions and help in facilitating the environmental monitoring of xenoestrogenic exposure. The selection of such marker genes relies on their sensitivity and specificity to the observed alterations at the morphological level. High throughput technologies and molecular biology offer reliable techniques for the detection of these physiological perturbations. Over the past decade, microarrays have been proven as efficient tools to determine the molecular mode of action (MOA) of environmental pollutants and to identify biomarkers as indicators of exposure for ecological risk assessments [10,11].

Since the establishement of molecular biomarkers, especially transcriptomic alterations, as early-warning signals of contaminants exposure, is needed in environmental monitoring programs, this approach is increasingly used in ecotoxicological studies, and named « (eco)toxicogenomic » [12]. Despite the gap between genomic knowledge regarding fish and mammals, several studies have used microarrays on fish (including orphan species) as model organisms, especially to monitor the impact of EDCs on gene expression patterns [11,13–16]. Microarrays are very powerful tools that can be used to measure the expression of thousands of genes or the whole transcriptome of an organism, in one single experiment, and the resulting expression profile can be compared under many experimental conditions. This technique has been widely used since the 1990’s and a considerable amount of data has been generated for an increasing range of species.

### 2.2. Statistical considerations

It is not simple to process datasets from microarray analyses due to the multiple levels of analysis and interpretation, from pre-treatment of the rough data to the filtering of statistically significant hits at the gene and gene-set levels. The different methods proposed and their different parameterisations can be combined into millions of analysis workflows that lead to different lists of significantly detected genes. While this technology offers great potential for generating data, several limitations should be considered during analysis of that data downstream, some of which are common to all fields of investigation and others that are more specific to surveys conducted on fishes.

First of all, as one statistical test is performed for each gene, thousands of tests are performed on a single array, which generates a very large number of false positive and false negative DEGs. Furthermore, the typically low numbers of replicates, given the cost of the technology, affects variance estimations. To circumvent the statistical limitations associated with the size of the datasets, the strictness of the threshold used to filter significant genes is increased as the number of replicates decreases [17]. However, a stricter threshold (type I error) decreases the power of the test (type II error), thereby increasing the rate of false negatives and limiting the detection of DEGs to those that are most obvious. Many authors have significantly improved the performances of such statistical analyses by solving methodological issues [18–20], and several performance benchmarks have been implemented to rank these methods [21–23]. However, the low positive and negative predictive power still remains a major issue [23].

Second, downstream data analysis and interpretation rely heavily on the availability/quality of knowledge resources for the genome of the species studied. In particular, the annotation of the probes and the amount of genomic knowledge, including ontologies and pathways, determine downstream data contextualisation. Nowadays, the constant evolution of genomic resources and the implementation of public databases have considerably improved the potential to infer and describe gene and protein networks, and shed light on their effect on patterns observed. Though such tools have been extensively used and validated in human-related genome-wide studies, the annotation/contextualisation of datasets regarding less studied organisms is more questionable. As an example, the full genomes of five fish species have been sequenced (namely zebrafish *Danio rerio*, stickleback *Gasterosteus aculeatus*, tetraodon *Tetraodon nigroviridis*, medaka *Oryzias latipes* and Fugu *Takifugu rubripes*) [24] and the sequencing of the genome of other species is still in progress (*e*.*g*. rainbow trout *Oncorhynchus mykiss*). However, genomic studies on fishes still suffer from the lack of a unified, exhaustive and validated source of knowledge compared to humans or rodents. The main bottleneck of the analysis/interpretation process is the quality of current genome annotations available for fishes. Nevertheless, we show here that existing tools can be adapted to fish, provided that their limitations are taken into account.

Third, besides statistical analysis issues, the biggest challenge in microarray data analysis is to cope with a large amount of data (several thousands of candidate genes) that often prevents validation of the results using other techniques, thus opening the way to too many hypotheses for further studies. Several visualisation techniques have been developed to aid in this analytical process, such as hierarchical and k-means clustering, principal component analysis or co-expression networks [25–27]. Among them, DAVID [28] performs a test derived from the Fisher Exact Test, called EASE. The enrichment p-value calculation (EASE-score) adapts the Fisher exact probability to the particularities of microarray data, with penalisation of the p-value for categories represented by only a few genes [29]. As highlighted by the growing number of dedicated software (i.e. DAVID/EASE [28,29], GoMiner [30], FatiGO [31], GenMAPP [32]), this approach is now currently used in microarray studies, due to its extraordinary potential to enhance the whole dataset analysis. However, the relevancy of absolute p-values remains questionable, as we will point out in the discussion section.

Though any of these methods alone can solve the statistic issue presented above when applied to experiments generating large lists of DEGs, we have described workflows [33–35] that progressively filter the output of the statistical analyses. The first goal is to focus on likely true positives in order to find meaningful biological signatures (stringent statistical thresholds and intersection of gene lists) and the second is to gather likely false negatives, in order to detect ontologies or pathways potentially involved (by relaxing the statistical thresholds and considering the union of gene lists).

### 2.3. Model organism

Rainbow trout is one of the most widely used fish species in ecotoxicology and constitutes a model organism in fish reproductive physiology. Sex determination is genetically controlled by a male heterogamety system (XX–XY), with a main male gene *Sdy* recently identified by Yano et al. [36]. Sex reversal occurs under massive exogenous hormone exposure. Moreover, in a previous study, we have shown that juvenile male rainbow trout developed testis-ova under chronic exposure to low doses of EE2 (10 and 100 ng/L). Measurement of the expression of key genes in gonads indicated that markers of testis development (*dmrt1* and *Sox9a2*) were down-regulated by the treatment, while the estrogen-responsive vitellogenin marker was strongly up-regulated [37]. Moreover, the rainbow trout genome is sequenced and a specific microarray is available [38]. Thus, a whole-genome analysis was done to gain insight into these mechanisms.

The goal of this study was to provide new insights on the feminisation induced by estrogenic substances in fish, at the transcriptomic level, through a microarray analysis of the gonad gene expression responses after chronic exposure to several doses of the potent xenoestrogen ethynylestradiol (EE2). Moreover, we aimed at offering new target genes as potential biomarkers of the ovotestis condition, to aid in the environmental monitoring of xenoestrogenic exposure in fish. To this end, we adapted a microarray analysis workflow that was previously validated on human cancer data [33–35] to toxicogenomic data using the rainbow trout as the model organism. This workflow included gene ontology and pathways mapping which are useful tools to retrieve biological meaning from large gene lists generated by microarray studies.

## Material and Methods

### 3.1. Ethics Statement

The experiment was performed according to European and national legislation for fish welfare and was approved by the University of Namur Ethics Committee (Agreement number LA 1900048; FUNDP consent 10/149).

### 3.2. Animals and hormonal treatment

The experimental method was previously described in details in Depiereux et al., [37]. Briefly, all-male rainbow trout (*Oncorhynchus mykiss*) fry were exposed from the onset of first feeding [Day 0 = D0 at 60 days post-fertilisation (dpf) to 136 dpf] to 5 nominal concentrations of 17α-ethynylestradiol (purity ≥ 98%, Sigma-Aldrich, Germany): 0 (solvent control), 0.01 μg/L, 0.1 μg/L, 1 μg/L and 10 μg/L, with 3 tanks per condition. The actual EE2 concentrations were measured in each tank at 6 time points using the Quantitative Ethynylestradiol Enzyme Immunoassay (EIA) Kit (Marloie, Belgium) according to the manufacturer’s instructions. Mean concentrations of EE2 ± SD were 0.08 ± 0.06 μg/l; 1.62 ± 1.74 μg/l and 9.88 ± 5.06 μg/l. The 0.01 μg/l EE2 concentration was under the detection limit (set at 0.02 μg/l). At the end of the exposure time, fish were anesthetized with MS-222 (140 mg/L) and sacrificed via incision of the spine. Thereafter, gonads were collected from all fish, immediately frozen in liquid nitrogen and stored at -80°C until RNA extraction. Gonads from 10 fish were pooled to reach enough material for further analyses.

### 3.3. Histological analysis

An histological analysis was previously made to investigate gonad morphological perturbations induced by the treatment [37]. Whole gonads investigation was done to make sure ovotestis can be detected by the observation of at least 6 transversal section planes taken over the entire gonad. Intersex gonads were found from the first concentration used (0.01 and 0.1μg/L), and complete sex reversal at the highest doses (1 and 10 μg/L). Based on the results obtained, gonads morphologies were classified into 4 classes as follows: testicular (gonads displaying the histological features of a differentiated yet immature testis), ovotestis (phenotype characterised by the presence of oocytes within the testis), and ovarian-like (gonads displaying the characteristics of an immature ovary). Several fish displayed an altered testicular morphology. These results are summarised in Table 1 (see results section).

**Table 1:** Summary of the histological results obtained in a previous study [37]. The results are presented as the percentage of gonad phenotypes observed in each concentration of EE2 tested. All the control fish displayed immature testes. Morphological disturbances were observed from the first concentration used (0.01 μgEE2/L) and complete sex reversal was observed from the 0.1 μgEE2/L concentration. Gonads morphologies were classified into 4 classes as follow: testicular (gonads displaying the histological features of a differentiated yet immature testis), ovotestis (phenotype characterized by the presence of oocytes within the testis), and ovarian-like (gonads displaying the characteristics of an immature ovary). Several fish displayed an altered testicular morphology.

Phenotype	EE2 concentration (μg/L)				
	0	0.01	0.1	1	10
Testicular	100	37	0	0	0
Ovotestis	0	48	40	0	0
Ovarian-like	0	5	30	89	100
Altered testicular	0	10	30	11	0

### 3.4. cDNA microarray experiment

The analysis was performed on a one-color 8x60K oligonucleotide array (Agilent Technologies: GPL15840) designed by the INRA-LPGP microarray platform (Beaulieu Campus, Rennes, France).

#### 3.4.1. RNA extraction and labelled cDNA synthesis

Total RNA was extracted from the gonads (n = 6; 2 pools of 10 pairs of gonads per tank, 3 tanks per condition) using TRIzol reagent (Invitrogen, Life Technologies Europe B.V., Ghent, Belgium) as described previously (Baron 2005). The total RNA concentration was determined using an ISOGEN NanoDrop 2000c spectrophotometer (Wilmington, Delaware, USA) and RNA quality was controlled on a Bioanalyzer 2100 (Agilent). Only samples with a RIN (RNA Integrity Number) >8 were kept for further analysis. Labeled cDNA (using Cyanine-3) was synthesised, purified, quantified and prepared for hybridisation following the Agilent protocol [38].

#### 3.4.2. Microarray hybridisation and raw data production

The samples were hybridised on the microarray slides with incubation at 65°C for 17 hours in a hybridisation chamber (Agilent). The slides were scanned and pre-processed (signal background corrections) using Agilent’s High-Resolution C Scanner. Raw data are available in S1 Dataset.

#### 3.4.3. Statistical analysis

The data analyses were performed using the R statistical software version 2.15.3. available on the R-Project repository (http://cran.r-project.org) and a set of packages available in the Bioconductor repository (http://www.bioconductor.org). Brief descriptions of the different steps of the analyses are provided below. Detailed scripts are presented in S1 Script.

The first steps were pre-processing and normalisation procedures, in which the expression values of the one-color microarray were first submitted to quantile-quantile normalisation using the normalize.quantile function in the preprocessCore package. Normalised data are available in S2 Dataset. Detailed script is available in S1 Script.a.

In a second step, an analysis of variance (ANOVA) was conducted on the normalized data. Variations in expression levels between replicate tanks (3 tanks per condition) were performed by pair-wise comparisons in R using the lmFit function in the Limma package available in Bioconductor (S1 Script.b.). We then performed a one-way ANOVA, with the EE2 concentration as the criterion, using the lmFit function of the Limma package (S1 Script.c).

The third step consisted on post-hoc comparisons. A post-hoc evaluation was performed using Scheffe’s method with the makeContrasts() and eBayes() functions provided in the Limma package (10 contrasts comparing all experimental conditions). The results were summarised using decideTests() and summary() (number of significant genes positively or negatively regulated for each evaluated contrast). The Benjamini-Hochberg procedure was used to adjust the p-values (correction for multiple tests) [39]. For each contrast, topTable() was used to extract top-ranked genes and relevant statistics (logFC, Average Expression, t statistic, p-value, adjusted p-value, B statistic) (S1 Script.d). More details are available in the R documentation file.

Following post-hoc comparisons, an analysis of intersections between selected contrasts was done. The set of common DEGs (S1 Script.e) between the top table of contrasts was defined using the intersect() function of the R package 'stat'. MAC OS 2011 (14.2.4) Excel software was used to select genes specific to a given intersection, by deleting those that were also differentially expressed in the other contrasts (see methodology for details).

The next step was devoided to the annotation of the array. There is no consensus for annotation of the Agilent 60K array. Bioinformatics tools have specific requirements with regards to gene identifiers (IDs) and traditionally need homogenous and unique identifiers. In addition, the annotation of nucleotides sequences continues to improve and can substantially differ within several months (regular updates). Therefore, the most recent annotation should be used to ensure the accuracy of the analysis. Ensemble gene IDs for *Danio rerio* (ENSDARG) were used to submit queries to the DAVID pathway analysis interface. The most recent gene symbols were used to analyse the data with EASE. The latest release of the trout INRA-Sigenae program (http://www.sigenae.org/, January 2013) was used to annotate the data using the UniProt [40] and e!Ensembl [24] databases.

When all DEGs with accurate annotation were obtained, a step of data visualisation was performed. We used a set of bioinformatics tools for the downstream analysis of differential expression to examine the biological context specific to the different levels of organisation (gene expression, pathways, ontologies), guided by the significance of the sets of DEGs tested. First, hierarchical clustering was performed using Cluster 3.0 (C clustering library 1.49) on the DEG highlighted by the statistical gene analysis (ANOVA) (available on http://bonsai.hgc.jp/~mdehoon/software/cluster/software.htm). The parameterisation of the hierarchical clustering was defined (i) to use the log-level procedure, (ii) to center the genes and arrays on zero, (iii) to cluster both genes and arrays, and (iv) to use the centroid linkage (average linkage) as the distance metric. The Java TreeView software (version 1.1.6r2) [41], available on http://jtreeview.sourceforge.net/ was used to visualise the results. A gene list was submitted to the tools available on the DAVID web interface (Database for annotation, Visualisation and Integrated Discovery)(http://david.abcc.ncifcrf.gov/home.jsp) [28,42], to identify and illustrate pathways defined in KEGG (Kyoto Encyclopedia of Genes and Genomes) as describes previously [33]. The background population was defined based on the genes targeted by the Agilent rainbow trout 60K array. A gene list was submitted to the EASE software (Expression Analysis Systematic Explorer), version 2.0 available on the DAVID website [29]. EASE is standalone software covering a wider range of gene identifiers (as compared to DAVID tools), and is able to handle the heterogeneous Swissprot annotation (orthologs from several species). Gene symbols were entered into EASE to proceed with the analysis of over-represented categories (the basic analysis was run on a non-redundant fully annotated data table). The quality of the analysis was illustrated in R with volcano-plots (p-value vs. fold-change).

The raw data and a normalised expression file are available through the accession number GSE58519 at the GeneOmnibus public data repository (http://www.ncbi.nlm.nih.gov/geo/).

#### 3.4.4. Validation of microarray data by real-time quantitative PCR experiment

Six genes among the oligonucleotides spotted on the chip, known as markers of early testicular development (*sox9a2*, *dmrt1*, *cyp11b*) or for their altered expression following estrogenic treatment (*vtg*, *esr1a*, *esr2b*) were selected for further analysis by real-time PCR, in order to validate the gene expression patterns obtained through the microarray approach. Real-time PCR analyses were performed as previously described [37]. Briefly, RNA was collected, purified, quantified, and stored as described in “RNA extraction” above (section 4.3.1.). Following extraction, samples were treated with DNAse (DNA-free kit, Ambion, Austin,USA) to avoid DNA contamination, following the manufacturer’s instructions. To obtain cDNA, 4 μg of total mRNA was reverse-transcribed using the RevertAid H Minus First Strand cDNA Synthesis Kit (Fermentas, Germany) according to the manufacturer’s directives. All samples and standards were compared with a negative reverse transcriptase control to ensure primer specificity (validation of CT values) and check we avoid genomic DNA amplification. Moreover, each plate contained water samples to serve as blanks. Specific validated primers were used for the 6 genes as described in Depiereux *et al* [37]. All primers were purchased from Eurogentec (Seraing, Belgium). Real-time PCR was performed in 20 μl (5 μl of cDNA, 2.5 μl of each primer at 500 nM, 10 μl MasterMix 2x) with SYBR Green (Applied Biosystems, Foster City, California, USA) as an intercalating agent. Each measurement was performed in duplicate. The PCR conditions were: 10 min at 9503B0043C, 40 cycles: 15 sec at 95°C, 1 min at 60°C. Relative quantifications were established by the comparative CT method (also known as the 2-ΔΔCt method) [43]. Relative gene expressions were calculated as the fold change in gene expression normalised to an endogenous reference gene (*hprt1*, previously validated for this experiment [37]) and relative to the untreated control (0 μg/L EE2), following these equations: ΔΔCt = (CT_Target_−CT_Housekeeping_)_Test_−(CT_Target_−CT_Housekeeping_)_Control_, and Fold change = 2^-ΔΔCt^. For the 6 genes tested, the correlation between differential gene expression generated through real-time PCR and microarray approaches was obtained by comparing mean values of 6 replicates per conditions in the 4 condition tested (the 4 concentrations; 0.01; 0.1;1;10 μgEE2/L). The significance threshold for n = 4 and p<0.01 is set to 0.72 [44].

## Methodology

In this section, we describe the specific development of the microarray analysis workflow to fit the particularities of the data collected. The workflow here, which was adapted from a workflow developed by our team on human Affymetrix platforms [33,34] and successfully adapted to another study [35] upon the same platform, was designed to handle a more complex experimental design (number of conditions) and the downstream annotation/pathway analysis which required that the specificities of fish species and the availability of genomic knowledge resources be taken into account.

Progressive filtering procedures were applied to the raw dataset obtained from a microarray study to restrict the size of the gene lists and to enrich them in true positives and biologically relevant DEGs (Differentially Expressed Genes). In summary, the workflow includes (i) the analysis of pre-treated data by (ii) clustering, then by (iii) one-way ANOVA followed by Scheffe post hoc pairwise comparisons. Thereafter, (iv) biologically relevant sets of contrasts and gene list intersections are selected at different levels of stringency. The remaining gene lists, which are supposed to be enriched in true positive DEGs, are (v) submitted to over-representation analysis methods (ORA). The workflow is summarised in S1 Fig.

- The raw data were pre-processed to remove biases due to technical errors and normalised to correct individual hybridisation signals. The first steps (including background corrections) are platform specific [45] and the Agilent technology provides accurate tools to improve the quality of the data from the raw output [46]. We then started with the gProcessedSignal data Agilent files. Normalisation is an important step that balances several replicates appropriately to allow for meaningful biological comparisons of expression levels. Several methods have been developed to normalise the data. We selected the quantile normalisation procedure that has been used extensively by the scientific community to scale datasets generated with one-color microarray platforms [47]. This method defines a shared distribution of mean intensity values computed from the quantiles of array-specific distributions of intensity values [48] and has been reported to ensure robust downstream statistical comparisons between arrays [47,49].
- The use of visualisation methodologies that sort the DEGs by expression profile, such as clustering methods [25] (see results), greatly helps reorder the dataset and focus on interesting groups of genes, but leaves the unsolved problem of low predictive power or even increases the risk of misinterpretation of the results. Moreover, in our analysis, the number of DEGs was often too high to successfully use this procedure, as the clusters identified displayed so many genes that it was impossible to treat them manually.
- As described above in the introduction, statistical analysis of microarray data is a non-trivial task and caution is required when performing this critical step. Most of the top-ranked methodologies involve optimisation of the variance estimation by sharing information across genes (i.e. the Shrinkage t [19], Windows t [50], Regularized t, Moderated t [51], etc.). In our case, the experimental design involved more than 2 factor levels (5 concentrations). To handle this design, we selected the Limma R package [51] that implements generalised linear models using an empirical Bayes model to assess moderated t and F statistics. Limma can be used to analyse both single and dual color microarray experiments. In addition, our previous benchmarks performed on a simulated dataset containing real data (biological variance) illustrated the quality of the results provided by Limma, which ranked second among 8 methodologies tested [23]. To increase the predictive power of the analysis, we conducted experiments with n = 6 in each condition. However, for n = 6 and for a fold change of -0.46 ± 3.86, approximately 80% true positives (i.e. 20% false negatives) were found in gene lists contaminated by 50% false positives [23]. Several filters were applied with varying stringency to focus on the more interesting genes (among the thousands of DEGs from the statistical output). The first filter operated on all DEGs revealed by the ANOVA was to apply Scheffe’s post hoc pairwise comparisons between the experimental conditions.
- To pursue the filtering, we selected sets of genes from the intersection of top gene lists, as reported by Pierre *et al*. on human cancer studies [33–35]. The principle of this approach was to improve the accuracy of the analysis by picking differentially expressed genes from intersections of gene lists obtained by pairwise comparisons. This strategy was applied on the list of DEGs inferred from the tested contrasts, thus the intersecting gene list that compares control and EE2-treated samples with the 0.01 μg/L and 0.1 μg/L concentrations (see Results section). Adjustment of the stringency of thresholds and intersections produced larger or shorter gene lists, depending on the bioinformatics tools used downstream, some of which take advantage of larger sets to minimise the number of false negatives, while others use smaller sets to minimise the number of false positives.
- The next step performed to further enrich the list of DEGs with true positives used over-representation analysis methods (ORA). These methods identify sets that are the most significantly observed in the top list of differentially expressed genes, compared with any other gene-set definition. Such sets of genes are defined based on criteria of interest for annotation and interpretation of the results (Ontology, Pathways, Transcription factor targets…). We previously used DAVID [28] to analyse the representation of pathways on cancer datasets and reported the discovery of the implication of several pathogen recognition pathways [33,34] and the spliceosome [35] in the metastatic phenotype under hypoxia. Enrichment analysis tools start with definition of the appropriate sets of genes (pathways, ontology,…), followed by comparison of (a) the number of member genes called differentially expressed for each gene set of interest with (b) the expected number of genes that would be detected by chance.

## Results

### 5.1. Hierarchical clustering on the whole dataset

**Fig 1:**
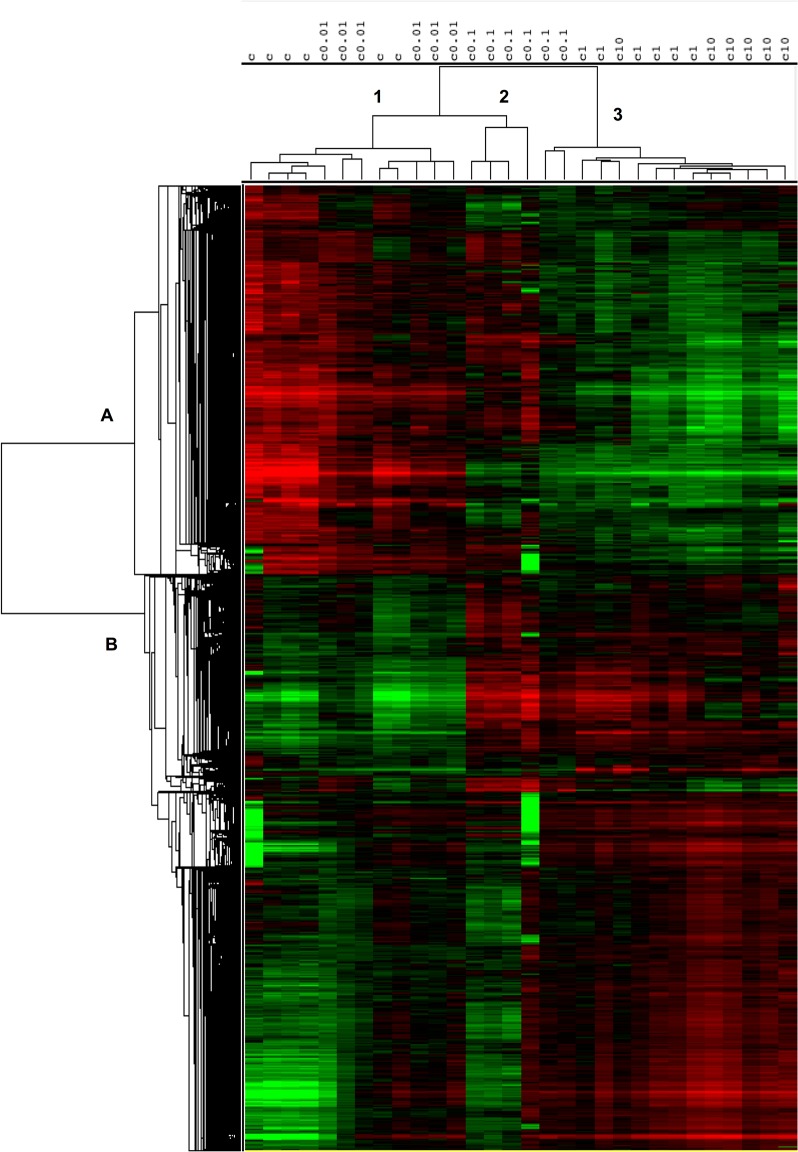
Hierarchical classification of gene expression profiles following steroid modulation of immature gonads in rainbow trout. This two-color matrix shows the 20,718 non-redundant differentially expressed genes in the testis of all-male rainbow trout following chronic exposure to 5 concentrations of ethynylestradiol (EE2) after statistical filtering by an ANOVA analysis using Limma (R software). c (control), c0.01, c0.1, c1 and c10 (respectively 0, 0.01, 0.1, 1 and 10 μgEE2/L). The genes (rows) and replicate arrays (columns) were classified according to their profile similarity, represented by branch lengths of the trees (increasing dissimilarity is shown with longer branches). Green represents under-expression, and red over-expression.

From the statistical analysis, 29,250 probes (20,718 non-redundant) appeared as differentially expressed (DEGs) in at least one tested condition (p < 0.05). In order to visualize these DEGs, a hierarchical clustering of the selected genes has been performed in Fig 1. It is interesting to note that EE2 concentration (columns) is associated with specific patterns of gene expression. By cutting the y-axis under the second level, we outlined three clusters: (i) the first cluster included the controls and the 0.01 μgEE2/L concentration; (ii) a second cluster grouped 4 samples of the 0.1 μgEE2/L concentration and (iii) a third contained two 0.1 μgEE2/L samples and all replicates at the higher concentrations (1 and 10 μgEE2/L). At the gene level (rows), two major clusters appeared with genes under or over expressed between the low (0.01, 0.1 μgEE2/L) and high (1 and 10 μgEE2/L) concentrations. As stated above, this huge gene list required further refinements in order to be interpreted.

### 5.2. Integration with disruptions observed at the morphological level (phenotypic anchoring)

**Fig 2:**
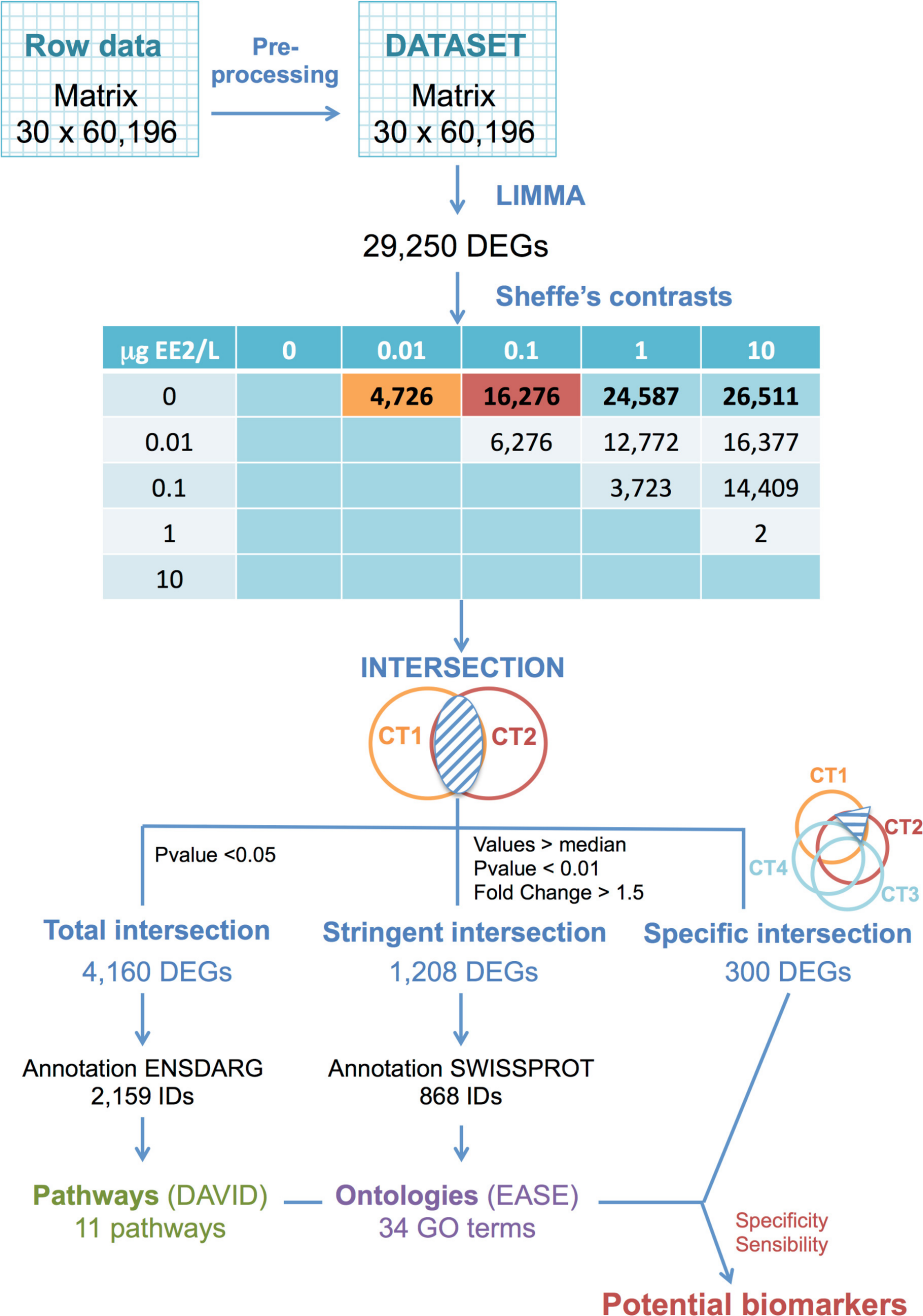
Flowchart of the workflow of the microarray analysis methodology and the associated results. The workflow includes analysis of pre-treated data by a one-way ANOVA followed by Scheffe post hoc pairwise comparisons, selection of biologically relevant sets of contrasts and intersection of gene lists, performed at different levels of stringency. The remaining gene lists, named “Total intersection”, “Stringent intersection” and “Specific intersection”, supposed to be enriched in true positive differentially expressed genes (DEGs) were subjected to over-representation analysis methods (ORA), namely pathways and ontologies research. To select potential biomarkers, we combined all approaches. We established three criteria from the “Specific intersection” group of genes: (1) specificity, (2) membership in a pathway/ontology, (3) sensitivity (high fold change). ENSDARG annotation refers to the ensembl database identifiers for *Danio rerio* genes.

The results obtained upon the histological analysis conducted previously [37] are summarised in Table 1. Gene expressions profiles acquired in the present study are consistent with these morphological disturbances. Indeed, major morphological alterations were observed from the first concentration used, with a high proportion of ovotestis gonads at the lower concentrations used (0.01 μgEE2/L), which is consistent with the high number of DEG retrieved between this condition and control fish (*i*.*e*. 4,726 DEG in Fig 2). Moreover, male phenotypes were also observed at this concentration, which may have been correlated with the mix of controls and [0.01] samples in the first cluster (Cluster 1 in Fig 1). Complete sex reversal was observed at the two higher concentrations used, all the fish displaying “ovarian-like” phenotypes at 1 and 10 μgEE2/L, which could be related to the third group of samples (Cluster 3 in Fig 1). The status of the 0.1 μgEE2/L dose was intermediary (fish displaying intersex and reversed gonads), which is supported by the intermediate pattern of gene expression in the cluster 2 (Fig 1). This approach allowed focusing our analysis on the two lower concentrations used, which were mostly represented by intersex fish.

### 5.3. ANOVA and Scheffe contrasts

Fig 2 summarises the microarray analysis workflow and shows an overview of the associated results.

First, we tested the effect of replicate tanks (3 tanks per condition) on gene expression. No effect could be detected (S1 Script. (b)), in such a way that differential expression analysis was performed using the one-way ANOVA assuming 6 independent samples for each condition (5 tested concentrations). The results for the 10 contrasts, presented by the number of differentially expressed genes without redundancy but before annotation, are shown in Fig 2. The cut-off for DEG inference was set to 0.05 for the adjusted p-values (Benjamini-Hochberg). The actual number of oligonucleotides having an accurate annotation was lower. An exhaustive list of the DEGs (both under- and over-expressed) is provided for each contrast in S1 Table. Considering the very large number of genes by contrast group (Fig 2), we later focused the analysis on four groups that were the most relevant with regards to the biological context, comparing reference samples with samples treated with EE2 at different concentrations. The contrast labels were defined as control vs. different [EE2]: (i) CT1 = Control VS [0.01 μgEE2/L]; (ii) CT2 = Control VS [0.1 μgEE2/L]; (iii) CT3 = Control VS [1 μgEE2/L] and (iv) CT4 = Control VS [10 μgEE2/L].

### 5.4. Selection of DEGs groups

As supported by the histological analysis of the gonads, we postulated that common DEGs detected in contrasts CT1 and CT2 should have contained the DEGs specific to the ovotestis process. The “Total intersection” group represented the set of DEGs that were common to CT1 and CT2, involving 4,160 oligonucleotides called differentially expressed (adjusted p < 0.05). We also defined a “Stringent intersection” set, which was expected to be enriched in true DEGs. Three arbitrary filters were applied (two prior to the statistical analysis, and a third one downstream of the statistical analysis). The first was based on the assumption that in a given tissue at a given time, only 50% of the transcripts are expressed. Indeed, the median signal intensity of a microarray is a commonly accepted estimation of the background and expression signals under this value can thus be considered as not expressed [52]. First, we only focused on genes for which the mean signal was up to the median in at least one experimental group. Second, to focus on transcripts with relevant regulation, we fixed a threshold of 1.5 for the fold change between tested samples. Last, we selected highly significant genes (p-value < 0.01). The resulting lists of DEGs built from both contrasts were called “Stringent intersection” and included 1,208 DEGs.

“Specific intersection” focused on DEGs that were specific to the CT1-CT2 intersection, which were expected to be specifically associated with the development of intersex features at the morphological level. We thus discarded those DEGs that were differentially expressed in the other contrasts (CT3 and CT4): 300 DEGs belong to this group.

**Fig 3:**
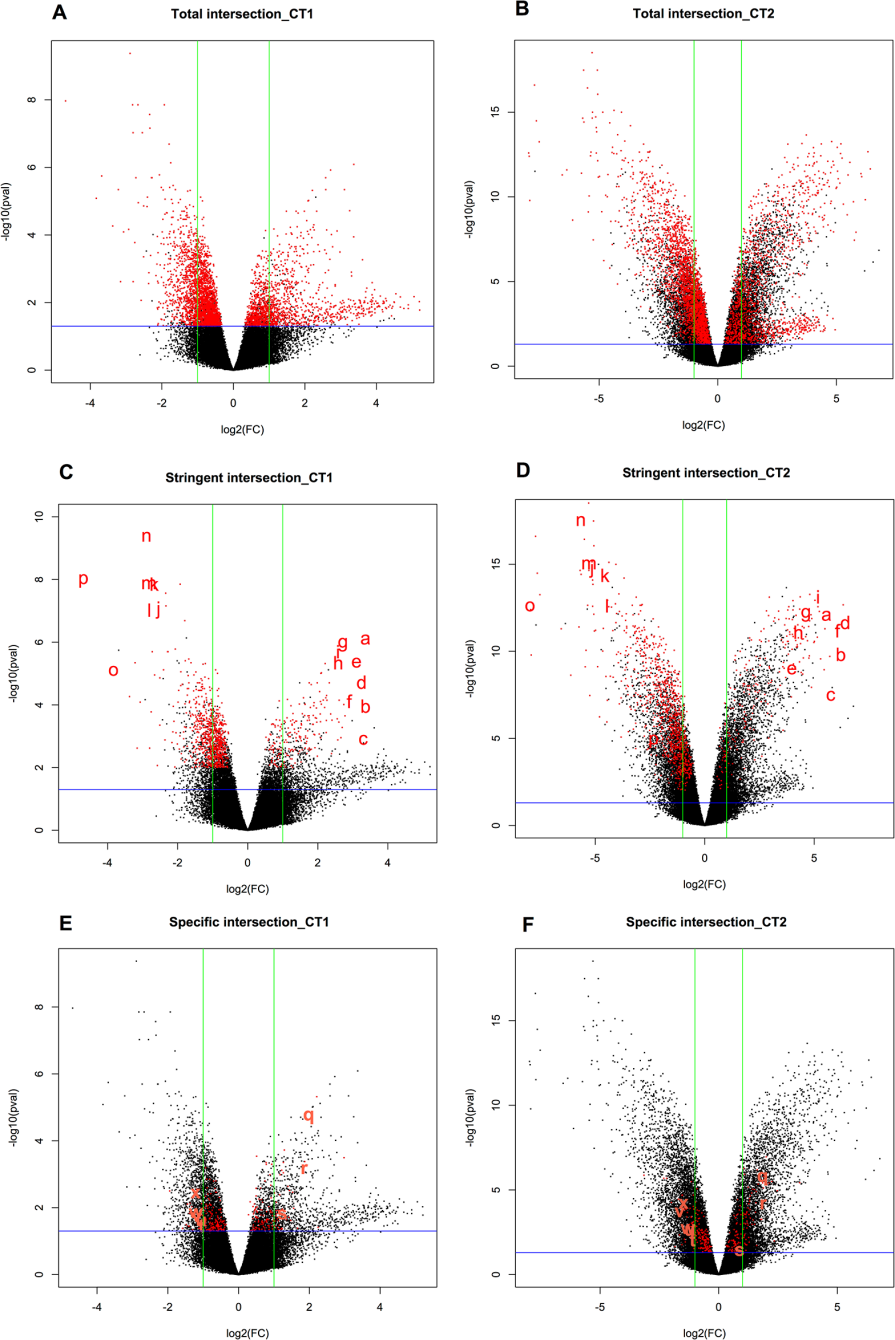
Volcano plots data representation. Volcano plots representing the 3 groups of DEGs selected for our analysis, in the two contrasts, CT1 (A-C-E) and CT2 (B-D-F). The green bars represent the log_2_(+/-2) fold change and the blue bars represent a p-value threshold of 0.05. The red dots are the 4,160 genes in the “Total intersection” group (A-B), the 1,208 genes in the “Stringent intersection” group (C-D) and the 300 genes in the “Specific intersection” group (E-F) selected at the intersection step. Letters correspond to the genes selected as potential biomarkers. a: *si*:*dkey-162h11*.*2*, b: Unknown, c: *THEG*, d: *LOC100136222*, e: *LOC795591*, f: *CU856539*.*4*, g: *thrap3*, h: Unknown, I: *dnaaf2*, j: *cyp2m1*, k: *Dnajc28*, l: *Bmp6*, m: *atp1a1*, n: *ADAR*, o: *he2*, p: *HES5*, q: *Foxl2*, r: *Spon2*, s: *cdkn1b*, t:*SLC25A6*, u: *Aldh7a1*, v: *TPl1*, w: *atp1a1*, x: *SLC25A4*. A complete description of gene names, their fold change and PValues are given in Table 5 for plots E-F (letters q to x) and Table 6 for plots C-D (letters a to p).

Volcano plots are provided in Fig 3 for the three groups of genes defined above (total, stringent and specific intersections) for each contrast to illustrate the selection applied to the dataset. This graph shows the results sorted by p-value (Y-axis) and fold change (X-axis). In such graphs, the most interesting genes are usually located in the upper left and right corners of the plot, depicting genes with low p-values and high fold changes. The comparison between the two contrasts helped to guide the selection of genes with interesting expression patterns at the two EE2 concentrations tested.

### 5.5. Over-representation analysis methods (ORA)

#### 5.5.1. Pathways

**Fig 4:**
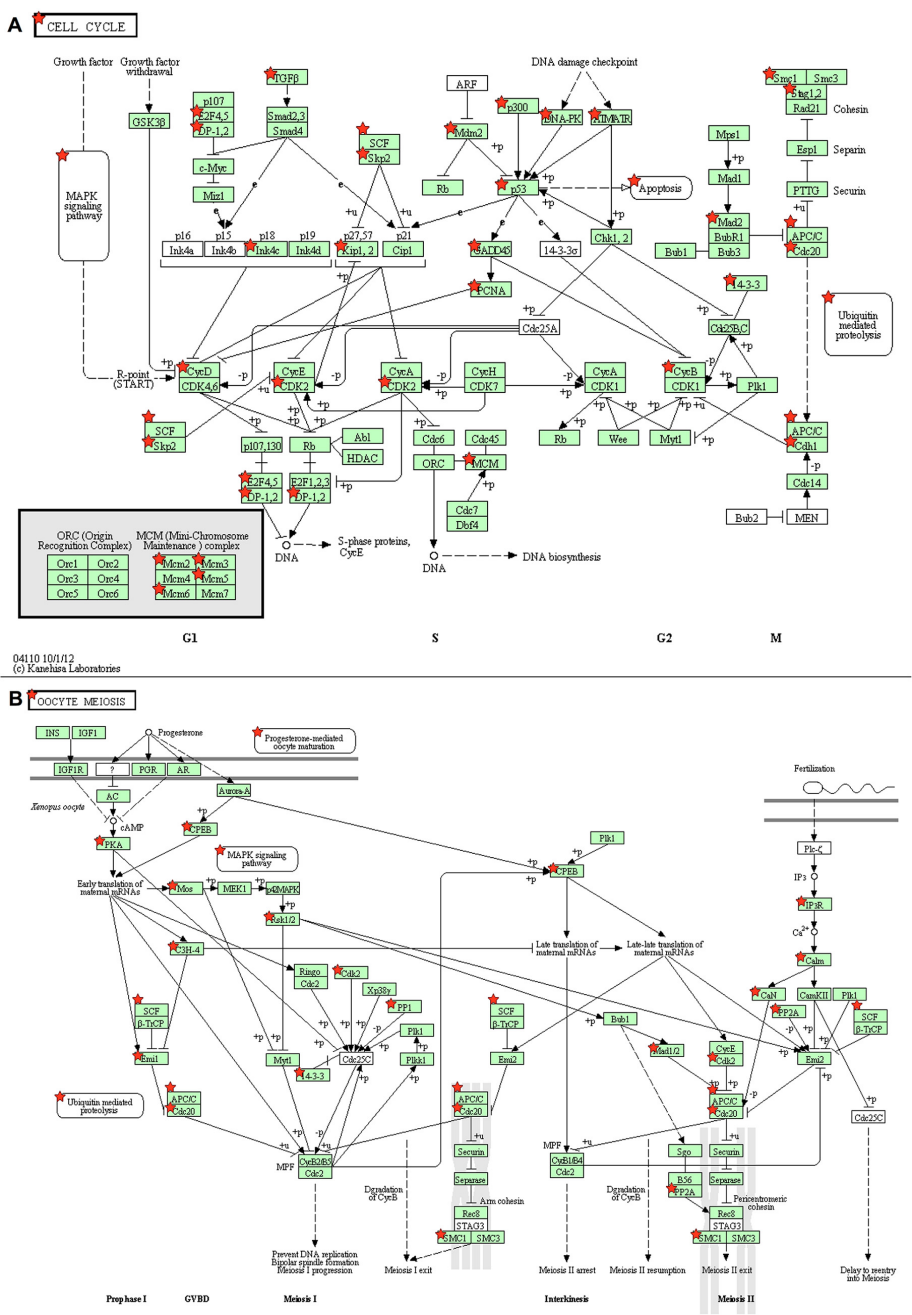
Examples of pathways resulting from the DAVID analysis. The Cell cycle (A) and Oocyte meiosis pathways (B)(in *Danio rerio*, retrieved from the KEGG database) are presented as examples of the results obtained in the pathway overrepresentation analysis made in this study. The red stars represent the DEGs in our results.

The list of Ensembl gene IDs for *Danio rerio* corresponding to the “Total intersection” DEG group was entered into the DAVID Web tool. Of the 4,160 DEGs, 2,412 were accurately annotated with an ENSDARG ID, and DAVID recognised 2,159 of them. The background was made up of 16,977 ensembl IDs. A total of 90 pathways containing at least 2 genes were identified from the remaining 2,159 genes (S1 Supporting Information). Among these pathways, 11 were detected with a significant enrichment in the list of DEGs submitted, with an EASE score below the threshold of 0.05 (Table 2). Only one pathway (the cell cycle) could be detected when the threshold was set to adjusted p-values, after the very conservative multiple testing Benjamini correction (see Table 2). Most of these enriched pathways involved Metabolism, and especially that of Carbohydrates (Glycolysis, Propanoate), Lipids (Fatty acid and Glycerolipid) and Amino acids (Valine, leucine and isoleucine degradation). Genetic information processing was also detected from the Spliceosome or in connection with the “Replication and Repair” processes. Of particular interest were the pathways related to cellular processes: (i) “Cell cycle” was the most significantly enriched set of genes, with the highest number of highlighted genes (37); and (ii) the “Oocyte meiosis pathway” was the third pathway in terms of the number of DEGs highlighted (24). The DAVID output for these two pathways is presented in Fig 4A and 4B). Gene enrichment is illustrated with red stars corresponding to the DEGs retrieved in our analysis.

**Table 2:** Results obtained for the pathways enrichment analysis approach. Significantly enriched pathways (EASE score < 0.05) retrieved by the DAVID web tool for the “Total intersection” group in this analysis (4,160 gene IDs restricted to a set of 2,159 well annotated differentially expressed genes recognized by ENSDARG identifiers in DAVID). This group represents the common genes differentially expressed following chronic steroid modulation of immature gonads in rainbow trout at 0.01 and 0.1 μgEE2/L. They were retrieved from the intersection between two contrasts (CT1 and CT2) represented by a majority of fish displaying intersex gonads.

Term	Count	P-Value	Benjamini
Cell cycle	37	2.1^E^-4	2.8^E^-2
DNA replication	15	8.9^E^-4	5.8^E^-2
Spliceosome	33	1.9^E^-3	8.2^E^-2
Valine, leucine and isoleucine degradation	15	6.9^E^-3	0.21
Glycerolipid metabolism	12	1.2^E^-2	0.27
Fatty acid metabolism	12	1.2^E^-2	0.27
Mismatch repair	9	1.4^E^-2	0.27
Glycolysis/Gluconeogenesis	18	1.6^E^-2	0.27
Propanoate metabolism	11	2.0^E^-2	0.29
Oocyte meiosis	24	3.0^E^-2	0.37
Nucleotide excision repair	13	3.4^E^-2	0.37

#### 5.5.2. Ontologies

An enrichment analysis was conducted on ontologies with EASE on the Gene symbol identifiers from the “Stringent intersection” DEG group. EASE identifies biological themes (GO Terms) from large gene lists [29]. Of the 1,208 DEGs in the Stringent intersection, 868 could be accurately annotated, and 410 were recognised by EASE. Interestingly, 77 terms were significantly enriched (EASE score lower than 0.05). The 3 main categories of the GO terms were not equally represented, with 65% belonging to the GO Biological Process, 23% to the GO Cellular Component and 12% to the GO Molecular Function. As in the pathway enrichment analysis, we hypothesized that the correction for multiple tests was too conservative and focused our attention on the p-values lower than 0.01 (highly significant genes). Table 3 provides a summary of the results obtained. An exhaustive presentation of the relationships between all genes and each category is provided in S2 Supporting Information. Genes that belonged to the “stringent intersection” set could be summarised in four broad terms with high significance: (i) intracellular including the more specific terms related to DNA replication and the nucleus; (ii) reproduction, involving genes known to take part in female gonad development; (iii) cell proliferation, with the more specific terms “morphogenesis” and “organogenesis”; and (iv) metabolic process which included the lipid metabolic process and more precisely genes involved in C21-steroid hormone biosynthesis.

**Table 3:** Results obtained for the ontology enrichment analysis approach. Significantly enriched GO terms (EASE score < 0.01) retrieved by EASE software for the “Stringent intersection” group in this analysis (1,208 gene IDs restricted to a set of 868 well annotated differentially expressed genes recognized by Gene Symbol identifiers in DAVID). This group represents the common genes differentially expressed following chronic steroid modulation of immature gonads in rainbow trout at 0.01 and 0.1 μgEE2/L. They were retrieved from the intersection between two contrasts (CT1 and CT2) represented by a majority of fish displaying intersex gonads, after a more stringent filtration of the dataset.

GO ID	Term	EASE score (P-Value)
GO:0005622	Intracellular	6^E^-4
GO:0008585	Female gonad development	7^E^-4
GO:0006730	One-carbon compound metabolism	7^E^-4
GO:0007049	Cell cycle	1.3^E^-4
GO:0000074	Regulation of cell cycle	1.4^E^-4
GO:0007276	Gametogenesis	1.5^E^-4
GO:0006261	DNA dependent DNA replication	1.7^E^-4
GO:0000003	Reproduction	1.8^E^-4
GO:0019953	Sexual reproduction	1.8^E^-4
GO:0005739	Mitochondrion	2.3^E^-4
GO:0005829	Cytosol	2.6^E^-4
GO:0000278	Mitotic cell cycle	4.4^E^-4
GO:0048232	Male gamete generation	5.0^E^-4
GO:0007283	Spermatogenesis	5.0^E^-4
GO:0008283	Cell proliferation	5.0^E^-4
GO:0005634	Nucleus	5.2^E^-4
GO:0006700	C21-steroid hormone biosynthesis	5.2^E^-4
GO:0008207	C21-steroid hormone metabolism	5.2^E^-4
GO:0005659	Delta DNA polymerase complex	5.3^E^-4
GO:0030894	Replisome	5.3^E^-4
GO:0016817	Hydrolase activity, acting on acid anhydrides	5.4^E^-4
GO:0008406	Gonad development	6.3^E^-4
GO:0009887	Organogenesis	8.5^E^-4
GO:0009653	Morphogenesis	8.8^E^-4
GO:0015207	Adenine transporter activity	9.2^E^-4
GO:0005345	Purine transporter activity	9.2^E^-4
GO:0001541	Ovarian follicle development	9.2^E^-4
GO:0046881	Positive regulation of follicle-stimulating hormone secretion	9.2^E^-4
GO:0042698	Menstrual cycle	9.2^E^-4
GO:0046887	Positive regulation of hormone secretion	9.2^E^-4
GO:0000502	Proteasome complex (sensu Eukarya)	9.5^E^-4

### 5.6. Real-time PCR validation

The accuracy of the microarray data was validated by means of real-time PCR on several genes which were shown to be up or downregulated in the microarray data, namely *dmrt1*, *sox9a2*, *cyp11b*, *vtg*, *esr1a*, *esr2b*. Q-PCR results obtained for the 6 genes tested are detailed in a previous paper [37], and summarized in S3 Supporting Information. The consistency of the results obtained by the two independent methods was tested by comparing the fold changes obtained by microarray analysis and real-time PCR in each condition (*i*.*e*. the four concentrations tested). The results revealed a very high correlation between both approaches for each gene tested, with R values much higher than the threshold set at 0.75 (for n = 4 and p<0.01)[44] (Table 4).

**Table 4:** Correlation between differential gene expressions obtained through real-time PCR and microarray approaches. Values were obtained from mean values of 6 replicates per conditions in the 4 conditions tested (the 4 concentrations; 0.01; 0.1; 1; 10 μgEE2/L). Significance threshold for n = 4 and p<0.01 is set to 0.72 [44].

Gene symbol	Description	Correlation coefficient
*dmrt1*	Doublesex and mab-3 related transcription factor 1	0.99
*sox9a2*	SRY-box containing gene 9b	0.99
*Cyp11b2*.*1*	cytochrome P450, family 11, subfamily b, polypeptide 2.1	0.98
*vtg*	vitellogenin	0.99
*esr1a*	Estrogen receptor α1 isoform	0.98
*esr2b*	Estrogen receptor β2 isoform	0.98

### 5.7. Biomarkers

This study resulted in the definition of two sets of potential biomarkers, respectively associated with different applications and scopes (see the Discussion). First, in the Specific intersection set, 237 out of 300 DEGs had a non-redundant accurate annotation, and 36 genes (of particular interest) belonged to at least one enriched pathway/GO term. Among the genes associated with more than one pathway/GO term, we selected the ones that were most significant. Considering that a high fold change will be more easily detectable (more sensitive biomarkers) only genes with (FC<-2 or FC>2) were selected from the CT1 contrast, corresponding to the lower dose. The resulting list included 8 DEGs as potential biomarkers of morphological disruption (Table 5). These genes are labeled with characters q to x in Fig 3 (E-F, volcano plots). The volcano plots showing the results for CT1 and CT2 highlight a high similarity between both contrasts (in term of fold change and p-value). *FOXL2* and *spon2* displayed the highest magnitude of over-expression in both contrasts (see Table 5 and Fig 3E and 3F). For additional information, the list of 29 DEGs with FC<-2 or FC>2 that were not associated with any enriched pathway/GO term are presented in S4 Supporting Information.

**Table 5:** Specific biomarkers. Potential “specific biomarkers” of morphological disruption (*i*.*e*. ovotestis) in juvenile male rainbow trout gonads exposed chronically to ethynylestradiol. These genes were selected following several filters to focus on genes specifically differentially expressed in the gonads of fish displaying intersex gonads. The “Graph symbol” column refers to the letters represented on volcano plots in Fig 3E and 3F.

Graph symbol	Gene symbol	Description	Fold Change CT1	PVal CT1	Fold Change CT2	PVal CT2	Pathway	GO term
**q**	*FOXL2*	Forkhead box protein L2 (*Homo sapiens*)	3.9	2^E^-5	3.6	2^E^-6	-	Intracellular
**r**	*spon2*	Spondin-2 (spon2), mRNA (*Salmo salar*)	3.6	7^E^-4	3.7	7^E^-5	-	Organogenesis
**s**	*cdkn1b*	Cyclin-dependent kinase inhibitor 1b (p27, kip1) (cdkn1b), mRNA (*Danio rerio*)	2.3	1.6^E^-2	1.8	4^E^-2	Cell cycle	Cell cycle
**t**	*SLC25A6*	ADP/ATP translocase 3 (*Homo sapiens*)	-2	3.4^E^-2	-2.1	8.8^E^-3	-	Intracellular
**u**	*Aldh7a1*	aldehyde dehydrogenase 7 family, member A1 (*Danio rerio*)	-2	2.5^E^-2	-2.3	2.6^E^-3	-	Valine, leucine and isoleucine degradation
**v**	*TPI1*	triosephosphate isomerase 1 (*Homo sapiens*)	-2.3	1.9^E^-2	-3.1	2.1^E^-4	-	Intracellular
**w**	*atp1a1*	ATPase, Na+/K+ transporting, alpha 1a.4 polypeptide (*Danio rerio*)	-2.3	1.4^E^-2	-2.4	2^E^-2	-	Hydrolase activity
**x**	*SLC25A4*	ADP/ATP translocase 1 (*Homo sapiens*)	-2.3	3.7^E^-3	-2.8	6.6^E^-5	-	Intracellular

Second, Fig 3 shows several strongly differentially expressed genes, presenting both a high fold change and a low p-value (upper right and left corners of the graph). From the “Stringent intersection” in the CT1 contrast (the lowest concentration used) (Fig 3B), we selected 16 genes (labeled from a to p) that are listed in Table 6. The same label was used in the volcano plot of the CT2 contrast (Fig 3C). In accordance with our definition of the “Stringent” and “Specific” intersections, genes belonging to this selection were common to CT1 and CT2 but also to at least one of the remaining contrasts, CT3 and/or CT4.

**Table 6:** Sensitive biomarkers. Potential “sensitive biomarkers” of morphological disruption (*i*.*e*. ovotestis) in juvenile male rainbow trout gonads exposed chronically to ethynylestradiol. These genes represent the most differentially expressed genes in term of fold change and p-value in fish displaying intersex gonads (i.e. the two contrasts CT1 and CT2, corresponding to fish exposed to 0.01 and 0.1 μg EE2/L against the control group, respectively). The “Graph symbol” column refers to the letters represented on volcano plots in Fig 3C and 3D.

Graph symbol	Gene symbol	Description	Fold Change CT1	PVal CT1	Fold Change CT2	PVal CT2	Path-way	GO term
**a**	si:dkey-162h11.2	si:dkey-162h11.2 [Source:ZFIN;Acc:ZDB-GENE-121214-90]	10.3	8.2^E^-07	47.42	9.2^E^-13	-	-
**b**	Unknown	-	10.3	1.1^E^-04	74.42	1.5^E^-10	-	-
**c**	THEG	similar to Testicular haploid expressed gene (Danio rerio)	9.8	1.3^E^-03	54.09	3.6^E^-08	-	-
**d**	LOC100136222	Oncorhynchus mykiss CD8 beta mRNA	9.5	1.9^E^-05	86.16	2.2^E^-12	-	-
**e**	LOC795591	similar to tubulin alpha 6 (Danio rerio)	8.6	4.5^E^-06	15.88	1.1^E^-09	-	-
**f**	CU856539.4	Uncharacterized protein (Danio rerio)	7.5	7.6^E^-05	66.91	6.5^E^-12	-	-
**g**	thrap3	thyroid hormone receptor associated protein 3b (Danio rerio)	6.6	1.2^E^-06	24.83	7.9^E^-13	-	-
**h**	Unknown	/	6.0	4.5^E^-06	19.54	7.9^E^-12	-	-
**i**	dnaaf2	dynein, axonemal, assembly factor 2(Danio rerio)	6.0	2.0^E^-06	36.40	7.4^E^-14	-	-
**j**	cyp2m1	Oncorhynchus mykiss Cytochrome P450 2M1	-5.8	9.4^E^-08	-35.37	1.9^E^-15	-	-
**k**	Dnajc28	DnaJ (Hsp40) homolog, subfamily C, member 28 (Danio rerio)	-6.4	1.4^E^-08	-23.47	4.3^E^-15	-	-
**l**	Bmp6	bone morphogenetic protein 6(Danio rerio)	-7.0	9.4^E^-08	-21.95	2.4^E^-13	-	Morphogenesis
**m**	atp1a1	ATPase, Na+/K+ transporting, alpha 1a.4 polypeptide (Danio rerio)	-7.1	1.4^E^-08	-38.90	1.0^E^-15	-	Hydrolase activity
**n**	ADAR	adenosine deaminase, RNA-specific (Danio rerio)	-7.4	4.3^E^-10	-50.37	3.3^E^-18	-	Nucleus
**o**	he2	hatching enzyme 2 (Danio rerio)	-14.2	8.2^E^-06	-251.12	2.6^E^-13	-	-
**p**	HES5	Transcription factor HES-5 (Homo sapiens)	-25.8	1.1^E^-08	-5.02	1.5^E^-05	-	-

**Fig 5:**
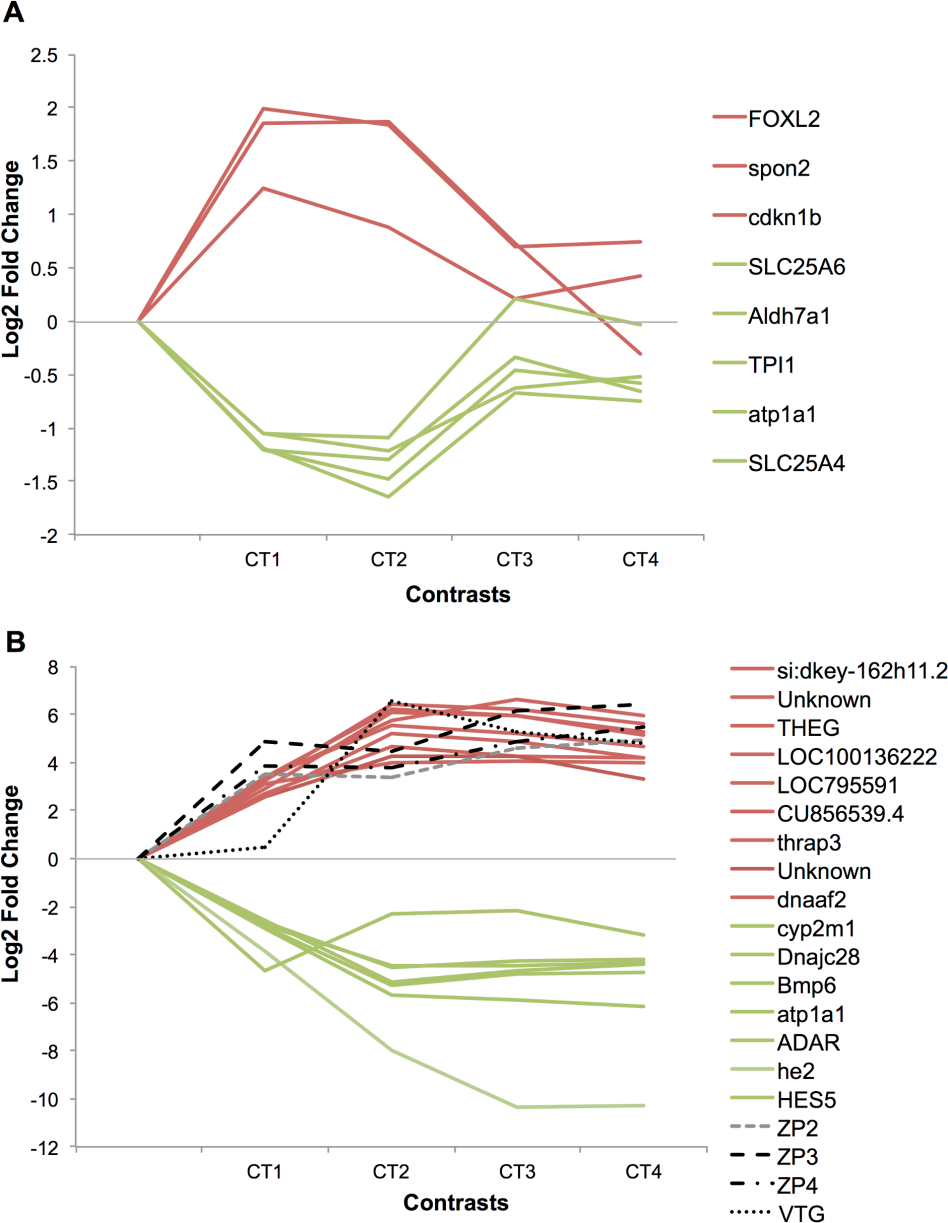
Potential biomarkers expression profiles on the whole experiment. Expression profiles of potential “specific” (A) and “sensitive” (B) biomarkers for the 4 contrasts of the experiments, expressed in log_2_ fold change. The control group was set to 0 (fold change = 1). A complete description of gene names is given in Table 5 for plots A and Table 6 for plots B.

The expression profiles of the two sets of potential effect biomarkers defined here from the study of the effects of EE2 at four concentrations are illustrated in Fig 5A and 5B). The two sets of candidate biomarkers displayed different patterns of expression. The first group includes genes with peak expression in the CT1 and CT2 contrasts, and weak or no expression in CT3 and CT4, whereas the second set contains genes for which the expression level increased at increasing EE2 concentrations.

## Discussion

### 6.1. Biological hypothesis

Overall, this study generated an impressive number of differentially expressed genes (DEGs), as highlighted by the hierarchical cluster (Fig 1) where nearly one half of the genes were significantly differentially expressed in at least one condition tested. The accuracy of the microarray data is validated by the similar pattern of expression obtained by QPCR analysis for the several selected genes, highlighted by the high correlation between both experiments results (Table 4). Moreover, the number of DEGs increased in a dose-dependent manner, as shown in the table of contrasts (Fig 2), with an increase in the number of DEGs as the EE2 concentration increased (CT1 to CT4). Thus, the expression of nearly one half of the content of the array was significantly altered at the highest concentration used (10 μgEE2/L) versus the control group (contrast CT4). This extraordinary effect may be related to the combination of several promoting factors: the maximisation of dose, duration and potency of the chemical used [12] and the complete reversal of genetic males into phenotypic females which can induce numerous changes at the transcriptional level [37]. Moreover, the juvenile state in fish is known to be more responsive to chemical treatment [53]. Considering this high number of DEGs in our analysis, it appears that clustering will not be the most suitable method to focus on genes of interest. Indeed, each cluster represented several thousands of genes, too many to handle manually, especially since this method does not resolve the problem of contamination of the data by false positives, which can impair the entire analysis step downstream.

Considering the second goal of this study which was to find potential biomarkers of morphological disruption occurring in fish gonads following chronic exposure to xenoestrogens, the combination of histological results obtained previously [37] with gene expression patterns allowed focusing our analysis on the two lower concentrations used (0.01 and 0.1 μgEE2/L), which were mostly represented by intersex fish. Due to the complete sex reversal observed at the higher concentrations used (1 and 10 μgEE2/L), gene expression observed may simply reflect differences between male and female phenotypes. This “phenotypic anchoring” approach, which combines results from different levels of biological organisation, has been proven to correlate the relationships between changes in gene expression and conventional toxicological endpoints [10,54]. Therefore, we hypothesized that the marker genes of the intersex condition must be present at the lower doses, which encompass a majority of fish displaying intersex gonads. These considerations, combined with the one concerning the statistical analysis of microarray data, have led to the use of a methodology aiming to focus on biologically relevant biomarkers, by choosing physiologically relevant sets of genes, and true positive enriched gene sets through the intersection of gene lists and ORA approaches.

The strength of this methodology is that it follows a step-by-step procedure, from general to more specific, depending on the bioinformatics tool used and using adapted lists of DEGs. Based on previous considerations, we choose CT1 and CT2 contrast intersection for the downstream analyses, using more stringent filter criteria which creates two groups of genes, named “Total intersection” and “Stringent intersection”. The use of DEGs common to these two contrasts focuses on a smaller group of genes representing potential genetic signatures of the intersex stage. The improvement of the dataset obtained between the “Total” and “Stringent” intersection groups of DEGs can be visualised in the Volcano plots (Fig 3AB–3CD), with a majority of the stringent DEGs being located on the edges of both the CT1 (Fig 3C) and CT2 (Fig 3D) graphs. This illustrates that DEGs selected with the CT1-CT2 intersection are relevant genes of the analysis (high fold changes and low p-values), and highlights a consistency of the DEG profiles between the two contrasts.

After these filtrations, the next step to further investigate these (still large) groups of DEGs was the use of ORA (over-representation analysis) methods. These publicly available methods identify meaningful information in large top lists. Ontology analyses [55] and more accurate pathway analyses are commonly used to rapidly extract biological meaning from large gene lists.

### 6.2. Inference by homology

As described above, rainbow trout was selected as the most suitable biological model for several reasons. At the time of data analysis the rainbow trout genome and complete functional annotation wasn’t available, though this is still not the case concerning the last one. We thus opted for interpretation by homology with *Danio rerio* for the pathway analysis, and with the accurate multi-species Swissprot annotation for the ontology analysis. This represented in itself an interesting challenge given the number of fishes still lacking genome sequencing and/or an accurate annotation. Our analysis was thus hampered by the lack of complete sequencing and annotation of the model species used.

### 6.3. Relevance of gene lists

Due to the limited genomic resources and annotation of the model species selected for our study, the interpretation and over-representation analysis raised several issues.

First, ORA results sorely depend on the annotation quality of sequences targeted by the array. To evaluate the enrichment of a set of genes, a comparison is performed with a null distribution (representation that can be expected by chance). The null distribution can be computed from the entire genome, or from a set of genes (*e*.*g*. genes spotted in the array). Distributions built from a larger number of genes allow for a better discrimination between significant sets (the p-values can be estimated more precisely) [28]. By default, DAVID uses the whole set of genes present in the genome as the background. This requirement is problematic when only part of the genome has been sequenced and annotated. Consequently, a homogeneous annotation must be performed by homology with a single species. The analysis of gene ontologies did not raise concerns since GO terms are not defined in a species-specific manner [55]. The analysis of pathways defined in KEGG (Kyoto Encyclopedia of Genes and Genomes) [56] was especially complicated: among fishes, only zebrafish (*Danio rerio*) has accurate pathways maps (exclusively in KEGG). The annotation of the Agilent rainbow trout 60K array was redundant and heterogeneous, such that we needed to unify the array annotation using *Danio rerio* orthologs, to ensure that DAVID could handle the associated gene IDs. We retrieved the *ensembl* gene IDs (ENSDARG) for this purpose [24]. This step constituted a significant unspecific filter and resulted in the loss of information due to the lack of correspondences, thus limiting the number of genes considered. To obtain more accurate results, we chose to set up the ENSDARG annotation of the array as the background for the pathway analysis by DAVID.

Second, the results of this analysis can vary greatly depending on the list of genes entered into the program, and especially its length. We conducted the over-representation analysis on several lists of genes detected with increasing stringency. We assumed that the pathway enrichment analysis required the larger gene list (with the lowest stringency) to have the chance of highlighting most of the genes in the same pathway, regardless of their p-value or fold change. The enrichment analysis of the ontologies was computed on a more specific set of DEGs (increased stringency) to avoid contamination of the results by too general terms. Last, to identify potential biomarkers, we generated a gene list with the highest stringency, and we combined the previously described approaches. For this, we established three criteria encompassing the two previous steps to select genes of interest: (1) specificity, (2) membership in a pathway/ontology, (3) sensitivity (high fold change) (Fig 2).

### 6.4. Statistical relevance

Despite the loss of information encountered during the annotation step, several interesting enriched pathways and GO terms were found in the DAVID and EASE analyses. However, another interesting aspect is the interpretation of EASE scores, as the multiplicity test correction (Benjamini) leads to virtually no significant output.

**Fig 6:**
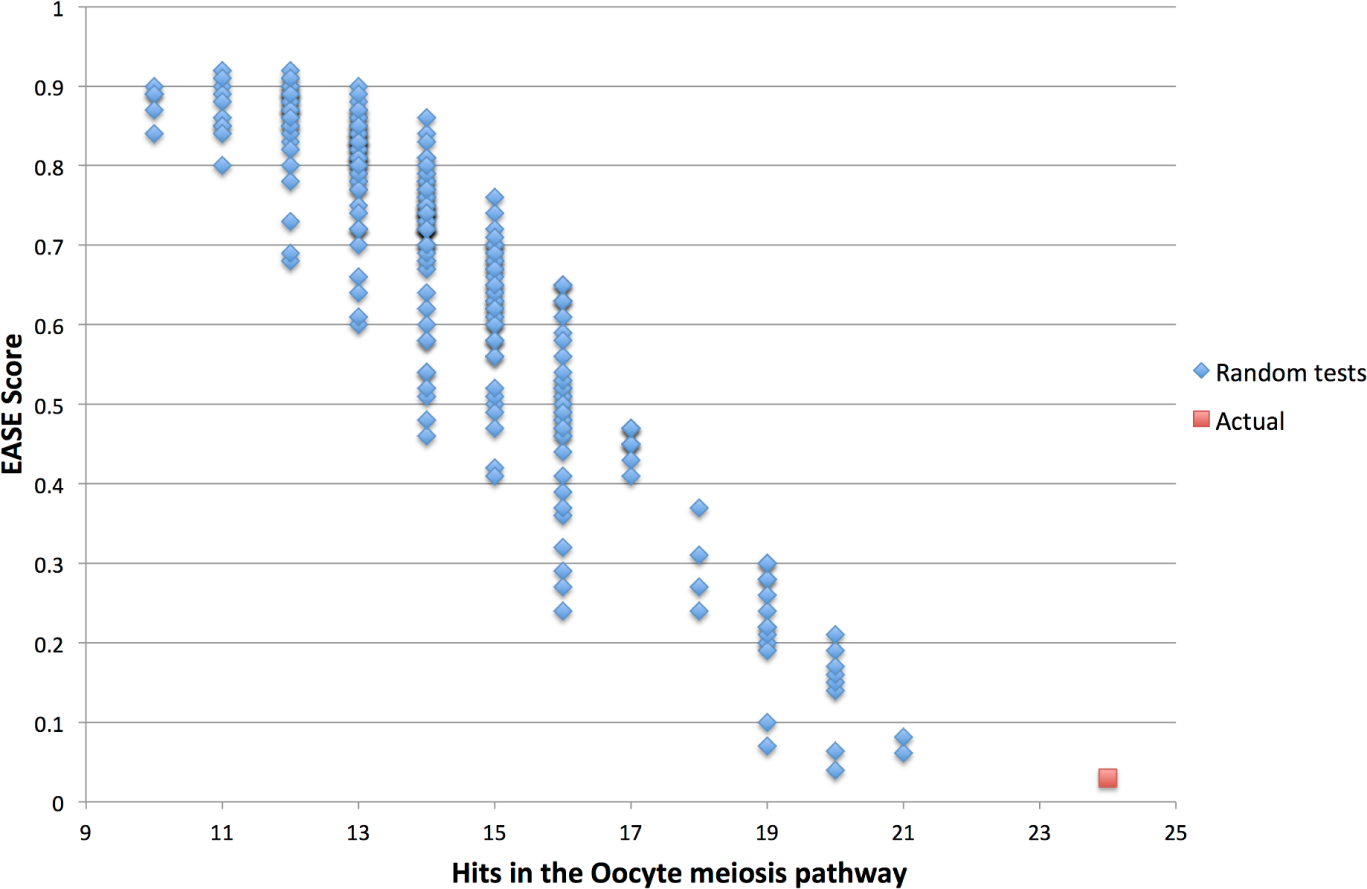
Null hypothesis for the Oocyte meiosis pathway. The plot represents EASE score and number of hits in the oocyte meiosis pathway for the 500 random selections from among the 2,412 gene identifiers (in blue), compared with the actual result of the analysis (red). This graph plots the number of hits (X-axis) against the EASE score (Y-axis). The difference between the random selection scores and the actual result score supports the assumption that oocyte meiosis is over-represented in our list of genes.

Indeed, care was required in interpreting the EASE scores (and the corrected p-values derived from it), according to their biological relevance in the context studied, the broadness of the information stored in the KEGG maps, the rate of false negatives induced by our screening and the obvious loss of information due to the inference by homology. The p-value is a controversial criterion to assess the truthfulness of a statistical result in microarray studies, especially at higher levels of analyses (*e*.*g*. pathway enrichment procedures) [57]. Moreover, the use of multiplicity compensation tests is also discussed, as their stringency increases with the number of tests performed in one experiment [57]. In the case of a microarray analysis, the number of tests computed is so large that the host of methodologies (FDR [58], FWER [59], Benjamini-Hochberg [39], …) has become too conservative. Therefore, they increase the false negatives generated, which reduces the overall information obtained. In his study, Konishi [57] attested that methods of correction for multiple testing are not suitable for microarray data, given the very high number of co-occurring tests. Indeed, the DAVID protocol states: “The analysis of large gene lists is more of an exploratory, computational procedure rather than a purely statistical solution” [28]. In our results, several pathways omitted by the Benjamini correction, such as oocyte meiosis (Table 2), seemed very interesting. To further assess the significance of the oocyte meiosis pathway in the over-representation results, we generated the null hypothesis by performing 500 random selections of 2,159 ENSDARGs among all the identifiers present on the microarray and entered them into the DAVID web tool. The EASE scores and number of hits in the oocyte meiosis pathway were then plotted (Fig 6). The plot clearly showed a gap between the random selections (with a maximum of 21 hits and an associated EASE score of 0.061) and the actual result (24 hits, EASE score of 0.03). More importantly, the same or better results were never obtained with 500 random tests, which estimates the probability of obtaining the results observed by chance at less than 0.01. The same work was done for the “cell cycle” pathway, with similar results and an estimated p-value below 0.01 (data not shown).

This simulation (Fig 6) clearly shows that these results departed from randomness, with even fewer random occurrences than expected by the EASE score (the probability of obtaining the results observed by chance being inferior to 0.01) and is corroborated by the similar results obtained for the “cell cycle” pathway (p <0.05 in DAVID with the Benjamini correction). This over-estimation of the EASE score by DAVID was also observed in previous studies [33–35].

### 6.5. Pathways and ontologies associated with testis-ova in fish

Results obtained in the ORA approach must be taken with caution regarding EE2 response and rainbow trout physiology. Indeed, pathways and ontologies retrieved in our experiment reflect the alteration in the transcriptome of intersex fish at one time point following a long-term exposure to low doses of EE2. Homology with *Danio rerio* genes was necessary since this is the only fish species for which pathways maps are available (see above). Considering this, pathways and GO Terms presented here are not biologically relevant of rainbow trout physiology given that correspondences between probes-to-genes-to-pathways between rainbow trout and zebrafish are not attested. However, many system structures, functions and regulations tend to be well conserved across all vertebrates [60]. Moreover, direct effect of EE2 cannot be ascertained after long-term exposure to the compound, given that indirect retro-control on the hypothalamus–pituitary axis or other systemic responses may also be involved in the response observed. Then, the pathways/GO terms obtained are considered as reflecting the fingerprint of the gene expression profiles in the testis of fish exposed chronically to low doses of EE2.

Interestingly, most of the pathways and GO Terms highlighted in our analysis has been previously associated with E2 and EE2 exposure using the rodent uterotrophic assay [61–63]. This assay rely on the sensitivity of the immature uterus to estrogens (by increased uterine weight) and has been widely exploited as a toxicological test for measuring the estrogenic activity of chemicals [64]. It includes pathways required for cell division (Cell cycle, DNA replication, Spliceosome, Valine, leucine and isoleucine degradation, Nucleotide excision repair, Mismatch repair, Glycolysis). All of these changes are consistent with the known physiological changes that occur upon exogenous E2-treatment [65]. Similar pattern has been also found after exposure to the phytoestrogen genistein and the synthetic xenoestrogen diethylstilbestrol [61]). The Cell cycle pathway was also found overrepresented (by pathways or GO Terms) in works studying the response of several fish species and several tissues (mainly gonads and liver) to estrogenic compounds [66–69]. The lipid metabolism (Glycerolipid, fatty acid) was also shown to be disrupted by EE2 in the zebrafish liver and testis [70,71]. Our results confirm that those pathways are a relevant fingerprint of the EE2 exposure, and even expended it to juvenile fish displaying intersex gonads. To our knowledge, the Oocyte meiosis pathway was never reported in previous studies. This alteration is consistent with the natural male versus female differentiation mechanisms considering that the over-expression of oocyte development is the earliest sign of female gonad development in fish [53]. The non species-specific Gene Ontology enrichment terms highlighted the same trends, with however more terms specifically related to ‘sexual reproduction’ (especially ‘female gonad development’, ‘gametogenesis’, ‘spermatogenesis’, ‘ovarian follicle development’), ‘steroid hormones production’ (‘C21-steroid hormone biosynthesis and metabolism’), ‘organogenesis’ and ‘morphogenesis’. Several ontologies such as the “mitotic cell cycle” and “nucleus” were also significantly enriched in juvenile androgen-masculinised female rainbow trout [72]. Despite the limitations encountered described above, the clear consistency of the results obtained in the ORA approach confirms the bioinformatics workflow performances to retrieve pathways and ontologies related to the context studied. Moreover, in addition to its utility for statistical purposes in the bioinformatics workflow (to retrieve true positives genes), these results reinforce the usefulness of this approach in highlighting biological meaning from large gene lists obtained in microarray experiments. Finally, these results argue in favor of the use of gene homology with *Danio rerio* to retrieve pathways related to exogenous estrogenic exposure, and suggest its generalisation to other fish species. This would greatly help for environmental risk assessment purposes.

### 6.6. Potential biomarkers

The final step of the analysis was to focus on potential biomarkers of morphological disruption occurring in fish gonads following chronic exposure to xenoestrogens. This will help greatly in environmental risk assessment procedures, by providing early-stage diagnostic tools for population exposure and effects. Following the previously described anchoring approach which considered that gene expression patterns in reversed fish (represented in the CT3 and CT4 contrasts) could be related to basic male versus female phenotype differences, we selected the DEGs specific to the intersection between CT1 and CT2 (“Specific intersection” group), and discarded the DEGs common to the other CT3 and CT4 contrasts. This step was performed to assess the specificity of the biomarkers and their use in a mixed fish population. To further increase the chances of true positive status, only genes belonging to a significant pathway/GO term were kept from this gene list.

Finally, to increase their sensitivity, only genes with a high fold change (at least over +/-2) in the lower concentration used (CT1) were selected. This resulted in a list of 8 potential specific and sensitive biomarkers of ovotestis occurrence in juvenile rainbow trout exposed to xenoestrogens (Table 5). Among them, the transcription factor *Foxl2a* is known as an early specific ovarian differentiation marker gene in rainbow trout [73]. The other genes encode enzymes or proteins involved in diverse cellular processes. However, though these genes were most probably biologically relevant in the context studied, their biomarker status seemed compromised as the size of their differential expression and p-value did not appear to be optimal. Indeed, as illustrated in the volcano plots (Fig 3E and 3F) only *Foxl2* (letter q) and *Spon2* (letter r) were located in an interesting position in the graph, whereas other genes were found at the upper edge of interesting dials (the upper right and left corners). One way to enlarge the pool of biomarkers retrieved by this study was to look at the genes mostly differentially expressed in the CT1-CT2 Stringent intersection. These genes were easily detected in the volcano plots and are presented as a second group of potential biomarkers in Table 6 and Fig 3C and 3D (letters a to p). Their sensitivity appeared to be suitable, but they were not specific as they were also differentially expressed for at least one of the other concentrations tested (CT3 and/or CT4).

The expression profiles at the four concentrations tested for the two groups of biomarkers selected further validated their different status (Fig 5A and 5B). Indeed, the methodology followed successfully retrieved genes with an expression peak in contrasts CT1 and CT2 for the first biomarker group, whereas only genes presenting a dose-dependent response were listed in the second group. A recent microarray analysis on Medaka testis-ova disrupted gonads after exposure to EE2 revealed up-regulation of genes related to zona pellucida (*ZP*) and the oocyte marker gene, *42Sp50* [13]. Using quantitative RT-PCR, they confirmed that the *Zpc5* gene can be used as a marker for the detection of testis–ova in male medaka. In our study, these genes and others known to be estrogen exposure biomarkers (*Vtg*, *CYP19a1*, *ZP* genes) or related to EE2 molecular modes of action (MOA) (*dmrt1*, *Sox9a2*, *amh*) [74,75] in the testis of several fish species were found to be significantly differentially expressed and were retrieved in the CT1-CT2 intersection (Total and Stringent intersection groups). However, when we looked at their expression profiles (for example *ZP* and *vtg* genes in Fig 5B), they appeared as not specific to the intersex condition. Therefore, genes belonging to the second group of potential biomarkers proposed in our study (Table 6) could potentially be specific effect biomarkers related to testis-ova development in rainbow trout. On the other hand, their dose-response pattern of expression could suggest that they are strong biomarkers of exposure to EE2. This could be determined by comparing these genes expression profiles in normal and estrogen-exposed females. It is worth noting that, as only one time point was analysed in our study, after a long period of exposure to the potent xenoestrogen EE2, these expression patterns could also reflect ancillary complex systemic responses to the treatment. Moreover, at the highest dose–duration combination, the observed changes in gene expression may no longer have been related to the mechanisms of toxicity specific to the contaminant [10]. Based on our results, the strong differential expression of these genes in terms of fold change and p-value suggests that they can be used at least under experimental conditions in mono-sex male fish populations, and could potentially be applied in natural conditions, with however the risk that they could also be expressed in females.

## Conclusions and Perspectives

In conclusion, we have successfully adapted a bioinformatics workflow to a toxicogenomic study on rainbow trout, a species that is not fully sequenced and annotated. At several steps of the analysis, the results support an enrichment of true positives in the set of DEGs selected. This procedure allows us to highlight several pathways significantly enriched with sets of genes differentially expressed in juvenile fish displaying intersex gonads after a chronic exposure to low doses of the potent xenoestrogen EE2. Most of these pathways are relevant of those previously related to estrogenic exposure, and interesting ones (*e*.*g*. the oocyte meiosis pathway) are also newly found in our experiment. Moreover, this analysis enabled us to propose several potential specific and/or sensitive biomarkers genes of testis-ova development in male rainbow trout for further validation in lab and field testing. Also, the data are ready to be re-analysed as soon as the rainbow trout genome functional annotation is available. Moreover, the use of significant Pathways/GO terms as criteria to choose these potential marker genes reinforced the chances of retrieving true positives in the analysis. Despite the lack of genomic information available for this species, relevant results were obtained by the ORA approach, taking into account inaccuracies due to the use of *Danio rerio* homologs for the probe annotation. This opens up the possibility of generalisation of this methodology in other fish species to search for the mechanisms underlying the male-to-female transdifferentiation process or on other (eco)toxicogenomic studies. For this, experiments involving both sexes, earlier developmental stages, lower concentrations and shorter exposure times are recommended.

## Supporting Information

S1 Dataset Raw data obtained from microarray analysis. (XLSX)

S2 Dataset Pre-treated data from microarray analysis. (XLSX)

S1 Fig Summary of the methodology followed for the microarray data analysis. Progressive filtering procedures were applied to the raw dataset obtained from a microarray study to restrict the size of the gene lists and to enrich them in true positives and biologically relevant DEGs (Differentially Expressed Genes).
(TIFF)

S1 Scripts Scripts (a-e) used in R software for microarray data analysis. (a) R script for data pre-processing and normalisation. (b) R script and results for inter-tanks effects. (c) R script for ANOVA analysis. (d) R script for contrasts analysis. (e) R script for intersection analysis.
(DOCX)

S1 Supporting Information Detailed DAVID output for the “Total intersection” group of DEGs in our microarray analysis. (DOCX)

S2 Supporting Information Detailed EASE output for the “Stringent intersection” group of DEGs in our microarray analysis. An enrichment analysis was conducted on ontologies with EASE on the Gene symbol identifiers from the “Stringent intersection” DEG group. EASE identifies biological themes (GO Terms) from large gene lists. Of the 1,208 DEGs in the Stringent intersection, 868 could be accurately annotated, and 410 were recognised by EASE. Interestingly, 77 terms were significantly enriched (EASE score lower than 0.05). The present excel file gives an exhaustive presentation of the relationships between all genes and each category.
(XLSX)

S3 Supporting Information Detailed QPCR results. Genes expression profiles of dmrt1, sox9a2, cyp11b, vtg, esr1a, esr2b, in the testis of juvenile rainbow trout chronically exposed to EE2, measured by Q-PCR.
(DOCX)

S4 Supporting Information Additional list of DEGs not associated with enriched pathway/GO term. (XLSX)

S1 Table Sheffe’s contrasts comparisons’ output from R software. (DOCX)
