# Supplementary figures and images for: Adaptation of a Bioinformatics Microarray Analysis Workflow for a Toxicogenomic Study in Rainbow Trout
Source: PLoS One. 2015 Jul 17;10(7):e0128598. doi: 10.1371/journal.pone.0128598 (PMC4506078; doi:10.1371/journal.pone.0128598)

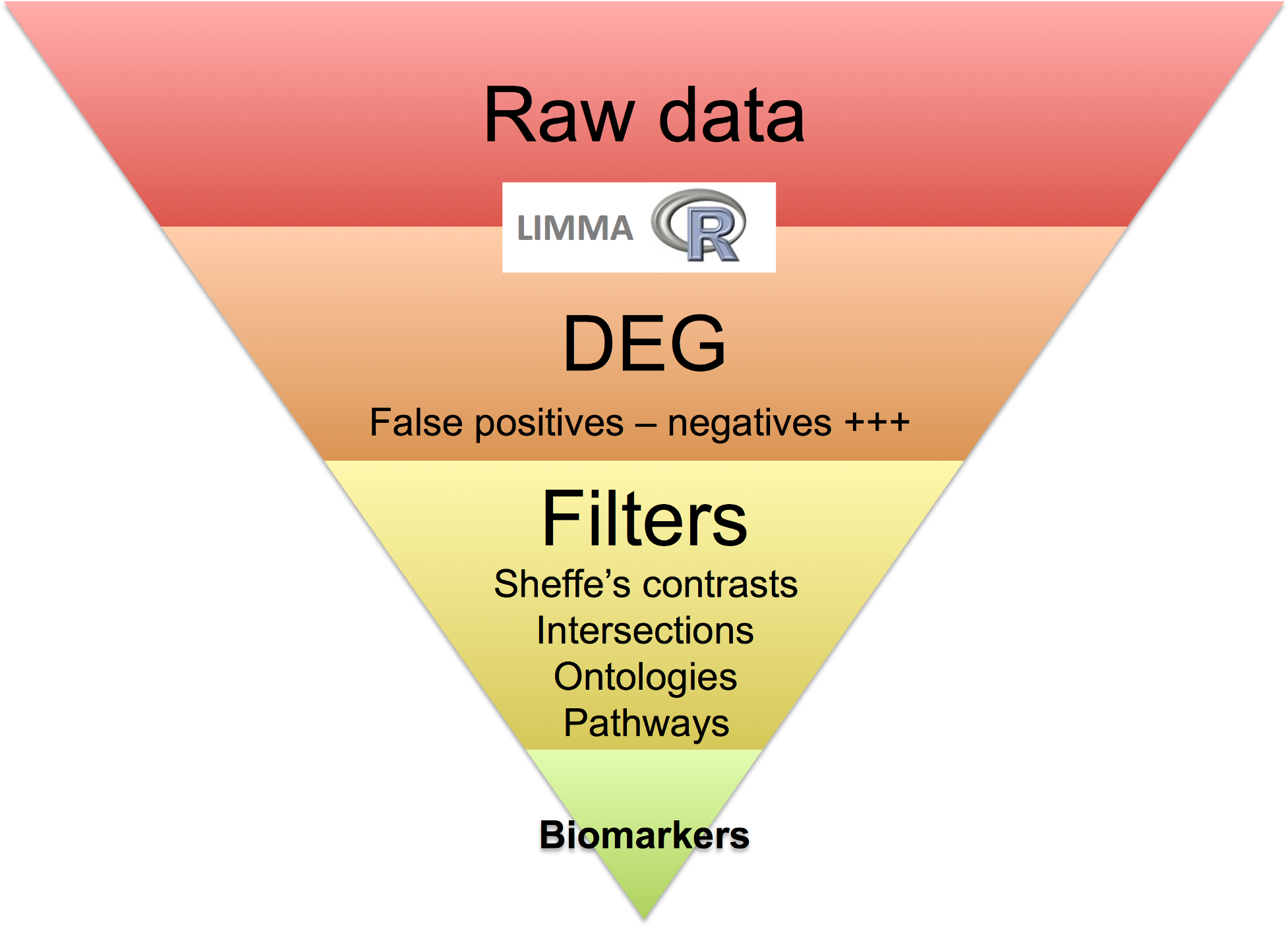

Supplement: S1 Fig — Progressive filtering procedures were applied to the raw dataset obtained from a microarray study to restrict the size of the gene lists and to enrich them in true positives and biologically relevant DEGs (Differentially Expressed Genes). (TIFF) [file pone.0128598.s003.tiff]
