# Supplementary material for: Adaptation of a Bioinformatics Microarray Analysis Workflow for a Toxicogenomic Study in Rainbow Trout
Source: PLoS One. 2015 Jul 17;10(7):e0128598. doi: 10.1371/journal.pone.0128598 (PMC4506078; doi:10.1371/journal.pone.0128598)
Supplement: S1 Scripts — (a) R script for data pre-processing and normalisation. (b) R script and results for inter-tanks effects. (c) R script for ANOVA analysis. (d) R script for contrasts analysis. (e) R script for intersection analysis. (DOCX) [file pone.0128598.s004.docx]

**S1 Scripts. Scripts (a-e) used in R software for microarray data analysis.** Scripts are in R language. Some objects and values, symbolyzed here by X or Y, have to be replace according to the dataset analyzed.

**a. R script for data pre-processing and normalisation**.

library(preprocessCore)

setwd("/ / ") # to set the folder of the csv file

sof<-read csv("DonnesGenCLEAN csv", sep=";")

exprs<-matrix(NA, ncol=(ncol(b)-1), nrow=nrow(b))

for (i in 1:nrow(c))

{

for (j in 1:ncol(c))

{

as<-as numeric(c[i, j])

exprs[i,j]<-as

as<-c()

}

}

exprs<- normalize quantiles(exprs)

rownames(exprs)<-b[, 1]

temp<-colnames(b)

temp2<-temp[2:length(temp)]

colnames(exprs)<-temp2

c<- b[, 2 :ncol(b)]

**b. R script and results for inter-tanks effects**. The control tanks 1 and 2 are used in this example. The script is in R language. The table summarize the results obtained for all the tanks interactions tested.

library(limma)

library(gcrma)

setwd("/ / ")

targets<-data frame(cbind(x=1:2, y=1:4))

targets$Filename<-rbind("Control 1A", "Control 1B", "Control 2A", "Control 2B")

targets$Target<-rbind("C1", "C1", "C2", "C2")

targ<-c()

targ<-targets$Filename

targ<-cbind(targ, targets$Target)

colnames(targ)<-c("Filename", "Target")

lev<-c("C1", "C2")

f<-factor(targ[,2], levels=lev)

design<-model matrix(~0+f)

colnames(design)<-lev

test<-exprs[,1:4]

fit<-lmFit(test, design)

result<-eBayes(fit)

topTable(result, number=20)

| Status | Control tanks | |  |
| --- | --- | --- | --- |
|  | C1-C2 | C1-C3 | C2-C3 |
| -1 | 12 | 1 | 1 |
| 0 | 60182 | 60193 | 60175 |
| 1 | 1 | 1 | 19 |
|  | [0.01] tanks |  |  |
|  | T11-T12 | T11-T13 | T12-T13 |
| -1 | 2 | 0 | 0 |
| 0 | 60193 | 60187 | 60193 |
| 1 | 0 | 8 | 2 |
|  | [0.1] tanks |  |  |
|  | T21-T22 | T21-T23 | T22-T23 |
| -1 | 0 | 4 | 8 |
| 0 | 60195 | 60190 | 60187 |
| 1 | 0 | 1 | 0 |
|  | [1] tanks |  |  |
|  | T31-T32 | T31-T33 | T32-T33 |
| -1 | 0 | 0 | 0 |
| 0 | 60195 | 60195 | 60195 |
| 1 | 0 | 0 | 0 |
|  | [10] tanks |  |  |
|  | T41-T42 | T42-T43 | T41-T43 |
| -1 | 0 | 2 | 0 |
| 0 | 60195 | 60193 | 60195 |
| 1 | 0 | 0 | 0 |

**c. R script for ANOVA analysis**. The targets are created to make the design of the analysis. The script is in R language. The design of the analysis is summarized in a table format. The numeric matrix exprs came from the script in the additional file 1 and contain normalized expressions.

- Design of the analysis

|  | **Tanks/Chip** | **Colnames** | **Target** |
| --- | --- | --- | --- |
| [0] | **1** | Control 1A | C |
| CTL | **1** | Control 1B | C |
|  | **2** | Control 2A | C |
|  | **2** | Control 2B | C |
|  | **3** | Control 3A | C |
|  | **3** | Control 3B | C |
| [0.01] | **1** | TEST1 1A | T1 |
| TEST1 | **1** | TEST1 1B | T1 |
|  | **2** | TEST1 2A | T1 |
|  | **2** | TEST1 2B | T1 |
|  | **3** | TEST1 3A | T1 |
|  | **3** | TEST1 3B | T1 |
| [0.1] | **1** | TEST2 1A | T2 |
| TEST2 | **1** | TEST2 1B | T2 |
|  | **2** | TEST2 2A | T2 |
|  | **2** | TEST2 2B | T2 |
|  | **3** | TEST2 3A | T2 |
|  | **3** | TEST2 3B | T2 |
| [1] | **1** | TEST3 1A | T3 |
| TEST3 | **1** | TEST3 1B | T3 |
|  | **2** | TEST3 2A | T3 |
|  | **2** | TEST3 2B | T3 |
|  | **3** | TEST3 3A | T3 |
|  | **3** | TEST3 3B | T3 |
| [10] | **1** | TEST4 1A | T4 |
| TEST4 | **1** | TEST4 1B | T4 |
|  | **2** | TEST4 2A | T4 |
|  | **2** | TEST4 2B | T4 |
|  | **3** | TEST4 3A | T4 |
|  | **3** | TEST4 3B | T4 |

targets<-data frame(cbind(x=1:2, y=1:30))

targets$Filename<-rbind("Control 1A", "Control 1B", "Control 2A", "Control 2B", "Control 3A", "Control 3B", "Test 1 1A", "Test 1 1B", "Test 1 2A", "Test 1 2B", "Test 1 3A", "Test 1 3B", "Test 2 1A", "Test 2 1B", "Test 2 2A", "Test 2 2B", "Test 2 3A", "Test 2 3B", "Test 3 1A", "Test 3 1B", "Test 3 2A", "Test 3 2B", "Test 3 3A", "Test 3 3B", "Test 4 1A", "Test 4 1B", "Test 4 2A", "Test 4 2B", "Test 4 3A", "Test 4 3B")

targets$Target<-rbind("C","C","C","C","C","C","T1","T1","T1","T1","T1","T1","T2","T2","T2","T2","T2","T2","T3","T3","T3","T3","T3","T3","T4","T4","T4","T4","T4","T4")

targ<-c()

targ<-targets$Filename

targ<-cbind(targ, targets$Target)

colnames(targ)<-c("Filename", "Target")

lev<-c("C","T1","T2","T3","T4") # the ANOVA analysis

f<-factor(targ[, 2], levels=lev)

design<-model matrix(~0+f)

colnames(design)<-lev

fit<-lmFit(exprs, design)

save(fit, file="fit.Rdata", compress=T) # fit contain data after the ANOVA

result<-eBayes(fit)

str(result$p value)

**d. R script for contrasts analysis**. The script is in R language. Some objects and values, symbolyzed here by x,y and z, have to be replace according to the contrast analyzed. The contrasts are set from the object design made in the additional file 3. The object fit came from the ANOVA analysis made in additional file 3.

cont<-makeContrasts("C-T1","C-T2","C-T3","C-T4","T1-T2","T1-T3","T1-T4","T2-T3","T2-T4","T3-T4", levels=design) # to analyse the contrasts

fit2<-contrasts fit(fit, cont)

results<-**eBayes**(fit2)

ber<-decideTests(results, p value=0 05)

summary(ber) # to print the summary table of the results (number of DEG over or under-expressed by contrast).

topTable(results[,1], adjust="BH", number=x) # the topTable function gives the results ranked in ascending order of the pvalues (adjusted with Benjamini Hochberg correction for multiple tests) of their differential expression, the number is set at 0.05.

topTable(results [,2], adjust="BH", number=y)

topTable(results [,3], adjust="BH", number=z)

ect…

**e. R script for intersection analysis**.

CT1<-read.csv("CT1 csv", sep=";") # To load the file containing the DEG of the contrast CT1

CT2<-read.csv("CT2 csv", sep=";") # To load the file containing the DEG of the contrast CT1

A<-intersect(CT1[,2], CT2[, 2]) # to select the common genes of these two groups
