# Supplementary material for: Adaptation of a Bioinformatics Microarray Analysis Workflow for a Toxicogenomic Study in Rainbow Trout
Source: PLoS One. 2015 Jul 17;10(7):e0128598. doi: 10.1371/journal.pone.0128598 (PMC4506078; doi:10.1371/journal.pone.0128598)
Supplement: S1 Supporting Information — (DOCX) [file pone.0128598.s005.docx]

**S1 Supporting Information. Detailed** **DAVID output for the Total intersection group in our microarray analysis**. The micrarray analysis was performed on juvenile raibow trout testis chronically exposed to several concentrations of EE2 (T1 to T4). The “Total intersection” group represented the set of differentially expressed genes (DEGs) that were common to the lower concentration of EE2 tested compared to the controls (CT1 and CT2), representing fish displaying intersex gonads. This group involved 4,160 DEGs (adjusted p < 0.05). Of the 4,160 DEGs, 2,412 were accurately annotated with an ENSDARG ID, and DAVID recognised 2,159 of them. The background was made up of 16,977 ensembl IDs. A total of 90 pathways containing at least 2 genes were identified from the remaining 2,159 genes.
