# Supplementary material for: Adaptation of a Bioinformatics Microarray Analysis Workflow for a Toxicogenomic Study in Rainbow Trout
Source: PLoS One. 2015 Jul 17;10(7):e0128598. doi: 10.1371/journal.pone.0128598 (PMC4506078; doi:10.1371/journal.pone.0128598)

**S3 Supporting Information.** **Detailed QPCR results.** Genes expression profiles of dmrt1, sox9a2, cyp11b, vtg, esr1a, esr2b, in the testis of juvenile rainbow trout chronically exposed to EE2, measured by Q-PCR. These results come from a previous article [37], referred to as Figure 9 A,D, E, F, G and I) and are summarized here. Graphs represent the relationship between fold change (expressed as mRNA relative expression ratio with control group) of differentially expressed genes and LOG[EE2] in the testis of rainbow trout fry exposed chronically to increasing concentrations of EE2. For each group, data represents the mean 6 2 SEM from 6 replicates measured independently. Each replicate consisted of a pool of 5 pairs of gonads. Letters a, b and c summarize the post hoc comparisons (p<0.05), the groups with the same letter being not significantly different. When the lack of fit to linear regression is not significant (p<0.05) the linear regression and associated R^2^ are shown.


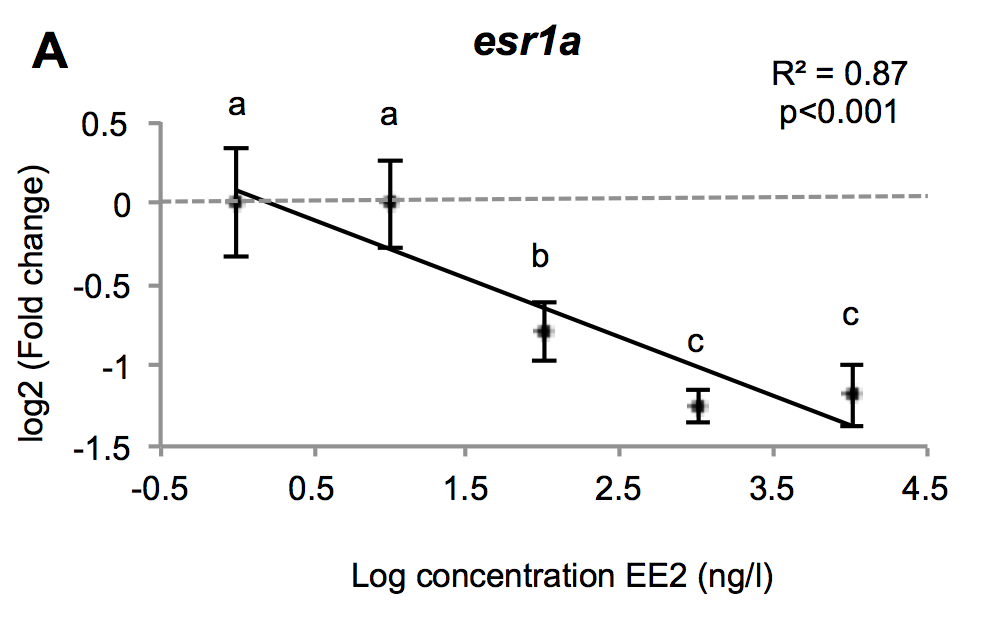

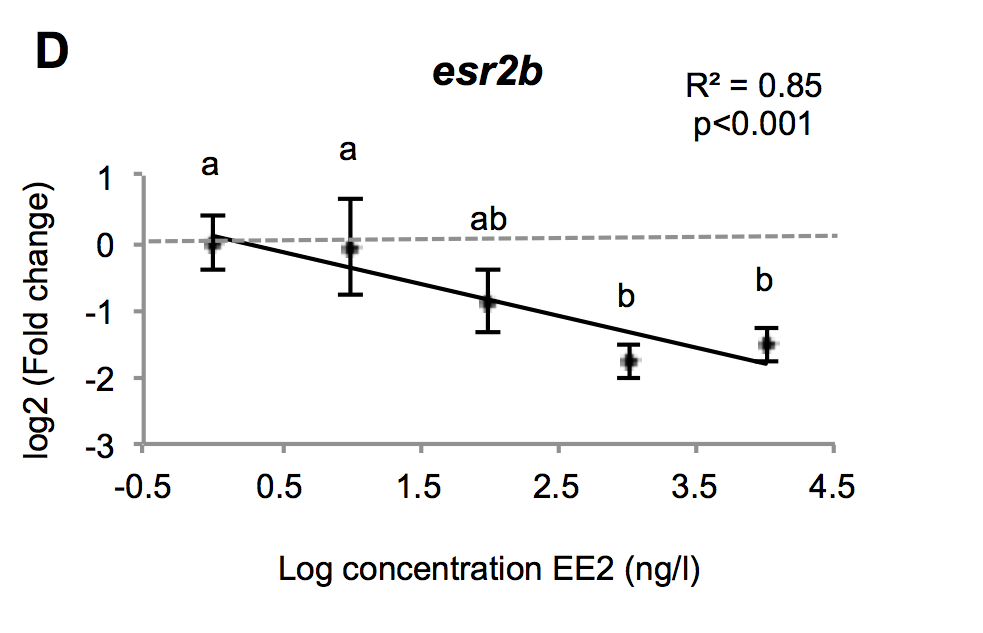


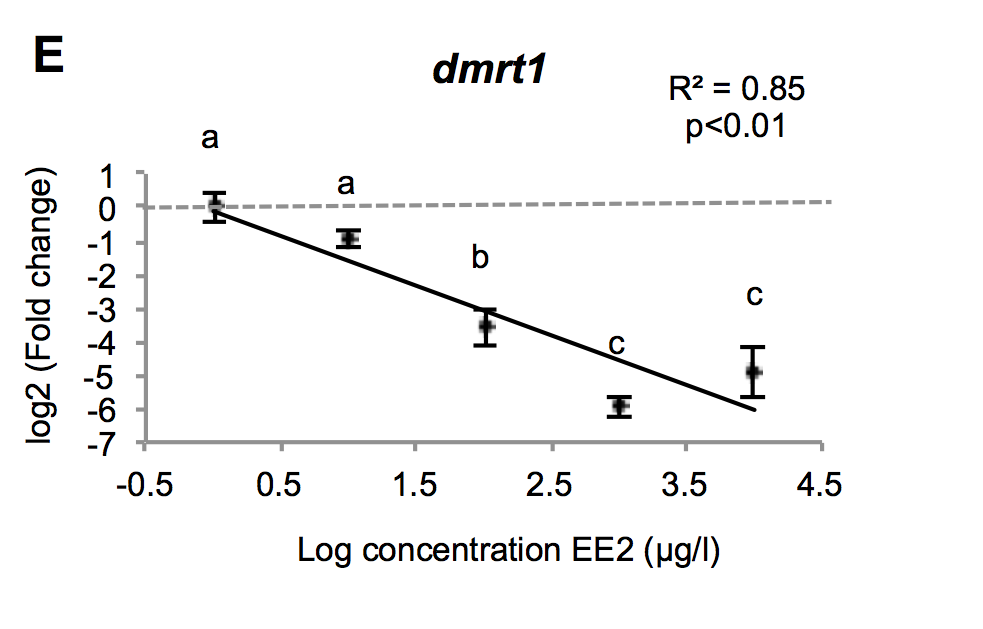

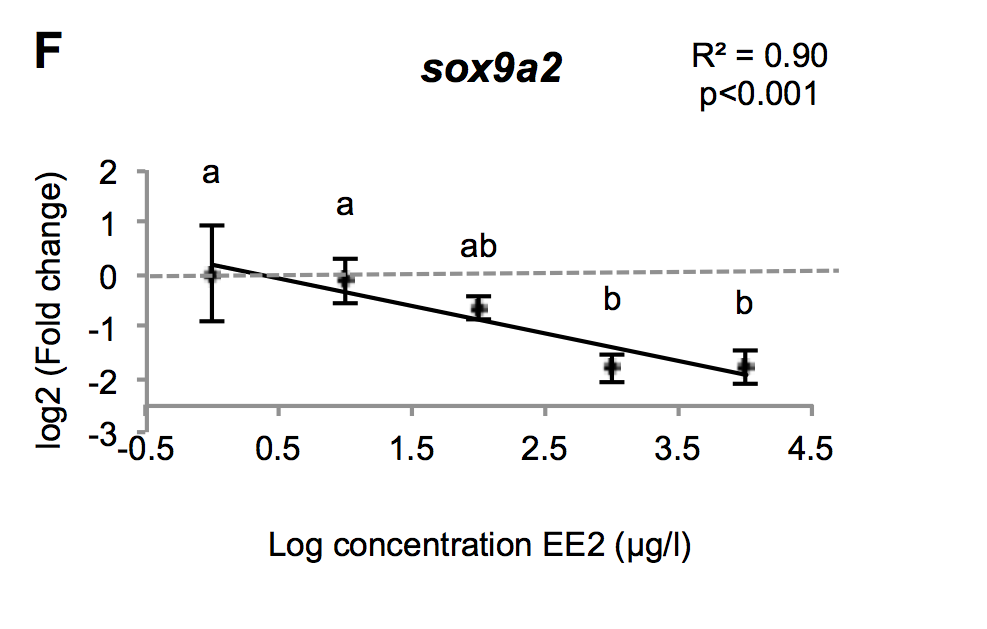


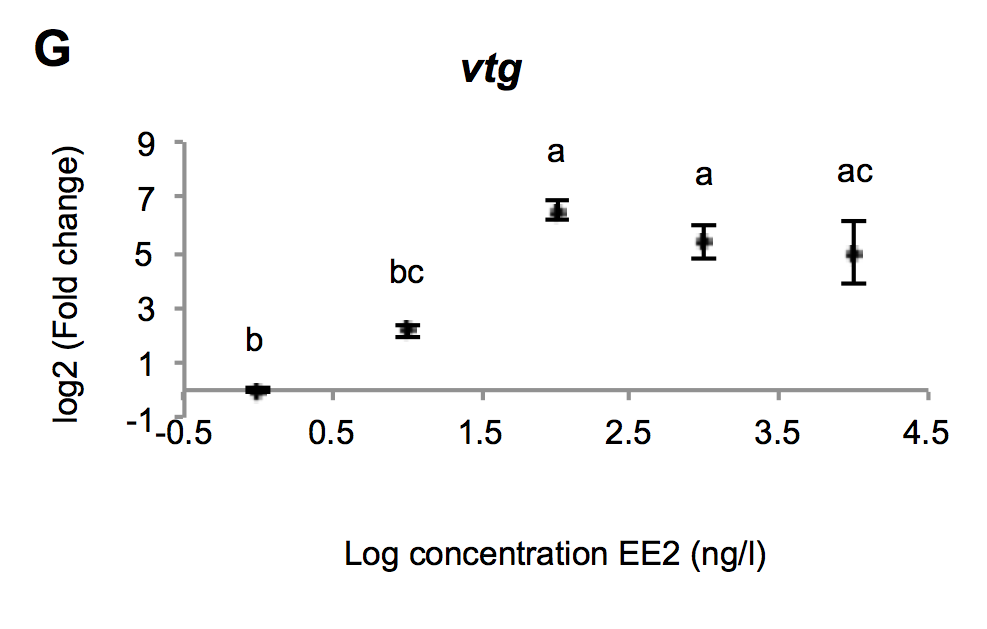

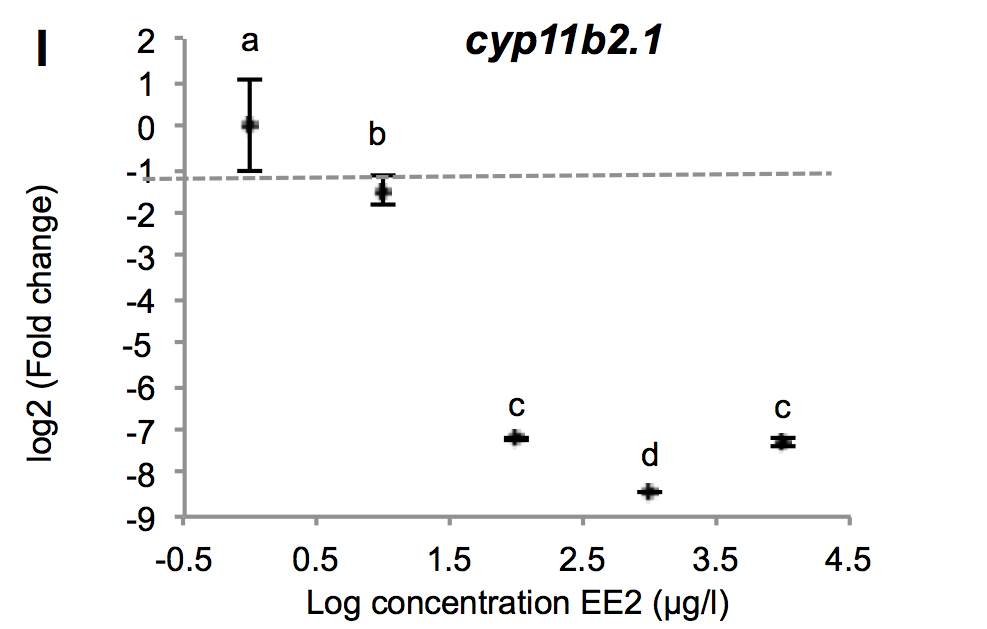

Supplement: S3 Supporting Information — Genes expression profiles of dmrt1, sox9a2, cyp11b, vtg, esr1a, esr2b, in the testis of juvenile rainbow trout chronically exposed to EE2, measured by Q-PCR. (DOCX) [file pone.0128598.s007.docx]
