# Supplementary material for: Adaptation of a Bioinformatics Microarray Analysis Workflow for a Toxicogenomic Study in Rainbow Trout
Source: PLoS One. 2015 Jul 17;10(7):e0128598. doi: 10.1371/journal.pone.0128598 (PMC4506078; doi:10.1371/journal.pone.0128598)
Supplement: S1 Table — (DOCX) [file pone.0128598.s009.docx]

**S1 Table. Sheffe’s contrasts comparisons’** **output from R software.** The table below provides d**etailed results (number of differentially expressed genes (DEGs), with redundancy) for the 10 pairwise Sheffe’s contrasts comparisons made in our microarray analysis.** C and CTL : control, T 1-4 : the four EE2 concentrations tested and their value in microgram per litre. Figures give the number of genes significantly differentially over-expressed (1), under-expressed (-1) and not differentially expressed (0) in testis of juvenile rainbow trout, between each experimental conditions compared.
